# Variance of human cortical synaptic transmission inferred from non-invasive magnetoencephalography of somatosensory evoked population spikes

**DOI:** 10.1016/j.isci.2026.117880

**Published:** 2026-10-20

**Authors:** Lukasz Radzinski, Rainer Körber, Jim Barnes, Soudabeh Arsalani, Benjamin Blankertz, Gabriel Curio, Gunnar Waterstraat

**Affiliations:** 1 Charité – Universitätsmedizin Berlin, Corporate Member of Freie Universität Berlin and Humboldt Universität zu Berlin, Department of Neurology, Neurophysics Group, Hindenburgdamm 30, 12203 Berlin, Germany; 2 Technische-Universität Berlin, Faculty of Electrical Engineering and Computer Science, Neurotechnology Group, Marchstraße 23, 10587 Berlin, Germany; 3 Bernstein Center for Computational Neuroscience Berlin, Unter den Linden 6, 10099 Berlin, Germany; 4 Physikalisch-Technische Bundesanstalt, Department of Biosignals, Abbestraße 2-12, 10587 Berlin, Germany; 5 Technische-Universität Ilmenau, Faculty of Computer Science and Automation, Institute of Biomedical Engineering and Informatics, Helmholtzpatz 5, 98693 Ilmenau, Germany

**Keywords:** high-frequency brain responses, neuronal population spikes, noninvasive magnetoencephalography, variability analysis, modeling

## Abstract

Non-invasive recordings of somatosensory evoked brain responses (SERs) contain high-frequency (∼600 Hz) evoked responses (hfSERs) which can be recorded at the single-trial level using ultralow-noise magnetoencephalography (MEG). hfSERs were shown to represent cortical population spikes and consist of an early (pre-synaptic) and a late (post-synaptic) component. Here, a statistical model of cortical population spiking reproduced single-trial hfSERs evoked by median-nerve stimulation in 5 healthy subjects. hfSERs were best replicated when variability was added only to the late (post-synaptic) model component, explaining 85% of inter-trial variability across subjects. Adding variability also to the early (pre-synaptic) component caused overfitting in most subjects, yet still indicated larger amplitude and latency variability post-synaptically. This proof-of-concept establishes model-based inference from non-invasively recorded population spikes as a means to estimate synaptic transmission variability in human neocortex. The approach opens a route to studying synaptic function in health and disease where invasive recordings are unavailable.

## Highlights

• Somatosensory Evoked Responses contain high-frequency activity >500 Hz (hfSERs)
• hfSERs are non-invasive correlates of cortical population spiking activity
• Modeling of hfSER records reveals larger post-than pre-synaptic hfSER variability
• Thus, the model estimates synaptic transmission variability from non-invasive data

## Graphical abstract

**Figure:**
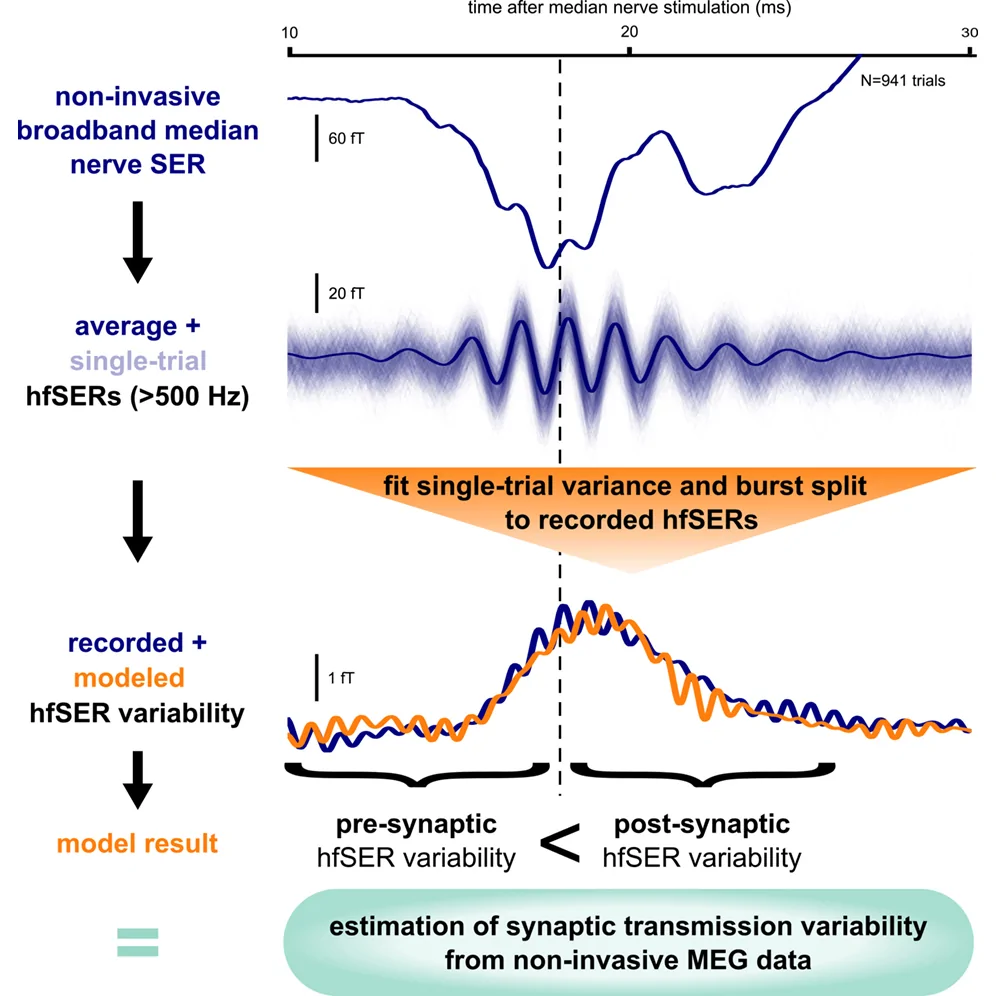

## Teaser

Health sciences; Medicine; Neurology; Medical imaging; Natural sciences; Biological sciences; Neuroscience; Techniques in neuroscience

## Introduction

Neuronal activity can be measured by recording its concomitant electric potentials or magnetic fields. Non-invasive measurements at the scalp level using electroencephalography (EEG) and magnetoencephalography (MEG) in the routine frequency range <100 Hz permit measurements of the slowly changing neural processes corresponding to post-synaptic dendritic or somatic potentials, i.e., neuronal input.^1^^,^^2^^,^^3^ Measuring single action potentials (i.e., neuronal output), on the other hand, commonly requires invasive sharp microelectrodes to be inserted in the brain.^4^^,^^5^^,^^6^

This methodological dichotomy effectively constrains measurements in healthy human subjects to recordings of neuronal input, while measurements of cortical spiking activity are mostly restricted to patients requiring brain surgery, such as in the case of a brain tumor, drug-resistant epilepsy,^7^^,^^8^ or deep brain stimulation for the treatment of psychiatric and movement disorders.^9^^,^^10^^,^^11^^,^^12^^,^^13^ Accordingly, there is a high need for an approach to record cortical spiking activity also in healthy subjects.

High-frequency activity (>500 Hz) in EEG and MEG recordings has been shown to correlate with cortical population spiking^14^^,^^15^ which provides an opportunity for noninvasive measurements in healthy subjects. A noteworthy example of high-frequency activity is high-frequency somatosensory evoked responses (hfSERs, “sigma bursts”)^16^^,^^17^^,^^18^ which reflect spiking activity of neuronal populations in the somatosensory cortex as revealed by concurrent invasive single-cell and epidural recordings.^19^ The ability to measure hfSERs non-invasively was demonstrated already starting in the late 1970s,^20^^,^^21^ and initial concerns whether hfSERs are technical or processing artifacts are meanwhile unequivocally overcome by demonstrating that hfSERs are not a filter artifact (Figure 1),^17^ have distinct intracranial generators,^16^^,^^22^ are stimulus-independently modulated,^23^^,^^24^^,^^25^ are not due to background brain oscillations^26^ and can also be elicited by physiological tactile stimuli.^19^ Measurements in non-human primates even confirmed that specific patterns of cortical spiking activity—measured with sharp invasive microelectrodes—were reflected in similar patterns of macroscopic high-frequency activity in epidural EEG recordings.^27^

**Figure 1:**
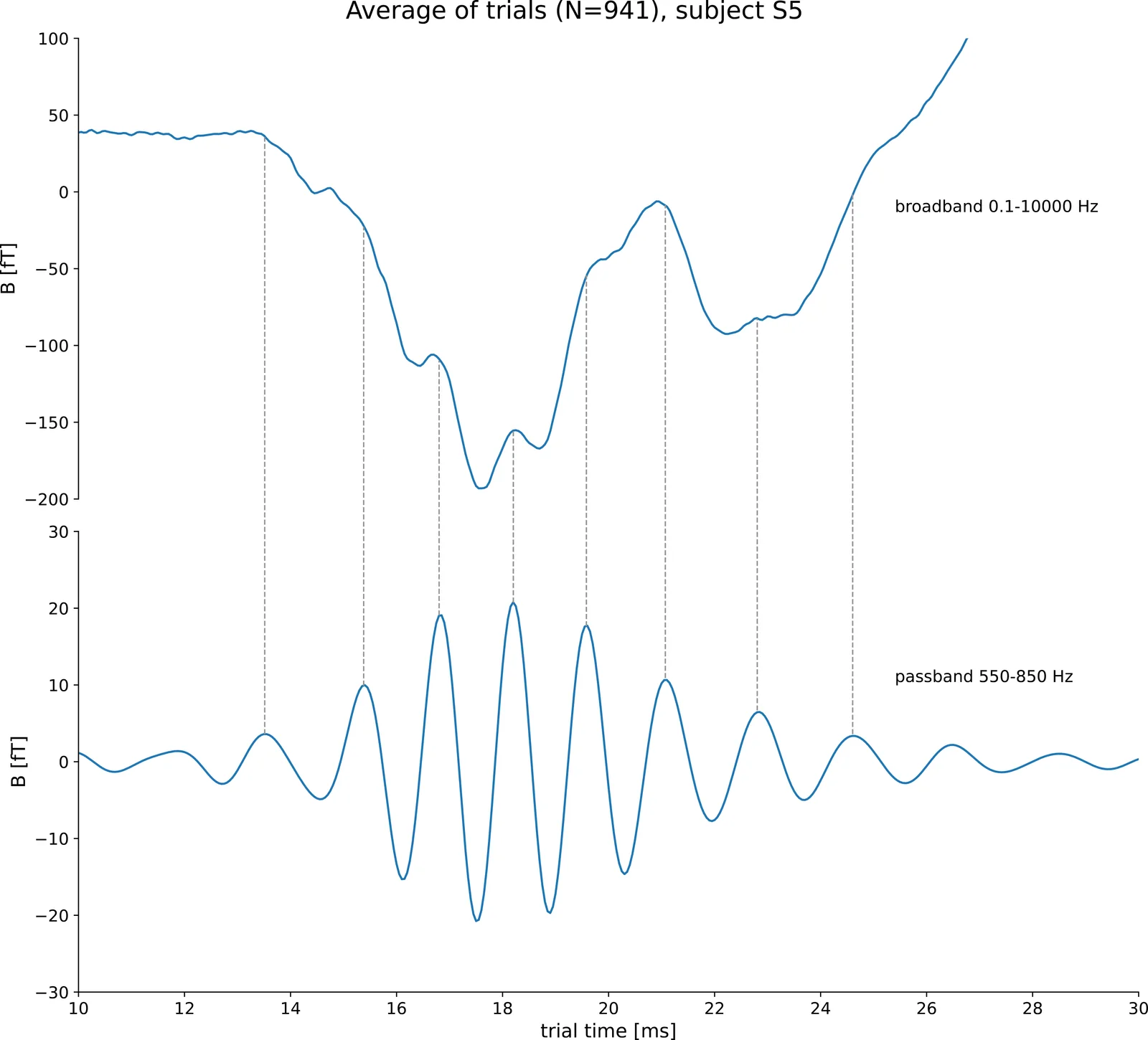
Averaged broadband (0.1–10000 Hz) and high-frequency passband evoked response (550–850 Hz) The broadband trace features a low-frequency N20 response (in this subject peaking at about 18 ms) superposed with a series of high-frequency notches and humps. These humps correspond one-to-one (dotted ancillary lines) to the peaks in the high-frequency passband signal (sigma burst) as obtained by latency-preserving digital filtering. Exemplary data from subject S5.

Furthermore, hfSERs were shown to consist of at least two components: an early burst component, originating from the thalamus and arriving in the primary somatosensory cortex as spike barrages traveling in the terminals of thalamocortical projection fibers, and a subsequent component generated in Brodmann area 3b of the somatosensory cortex.^16^^,^^22^^,^^28^ The rapid depression of the late burst component in the face of increasing stimulation frequency,^29^ as well as its selective suppression after the application of the synaptic blocking agent kynurenic acid to the cortex^30^ demonstrated its postsynaptic origin, while the early (pre-synaptic) burst component remained unchanged.

The main limitation of this approach, until recently, was the low signal-to-noise ratio (SNR) of neural high-frequency activity >500 Hz, the amplitude of which usually falls below the noise floor of standard EEG and MEG, necessitating averaging and therefore precluding the analysis of single-trial hfSER variability.^19^^,^^31^ To resolve this limitation, dedicated low-noise EEG^32^^,^^33^ and MEG systems^34^ have been designed and optimized for single-trial high-frequency activity measurements. A critical threshold was crossed when a single-channel ultralow-noise MEG system (white noise level 0.18 fT/√Hz)^35^ enabled a signal-to-noise ratio sufficient not only to detect^36^ and observe single-trial hfSERs (Figure 2, “rec.”) but even to depict the inter-trial variability^37^ of hfSERs that were evoked by repetitive median nerve stimulation in healthy subjects.

**Figure 2:**
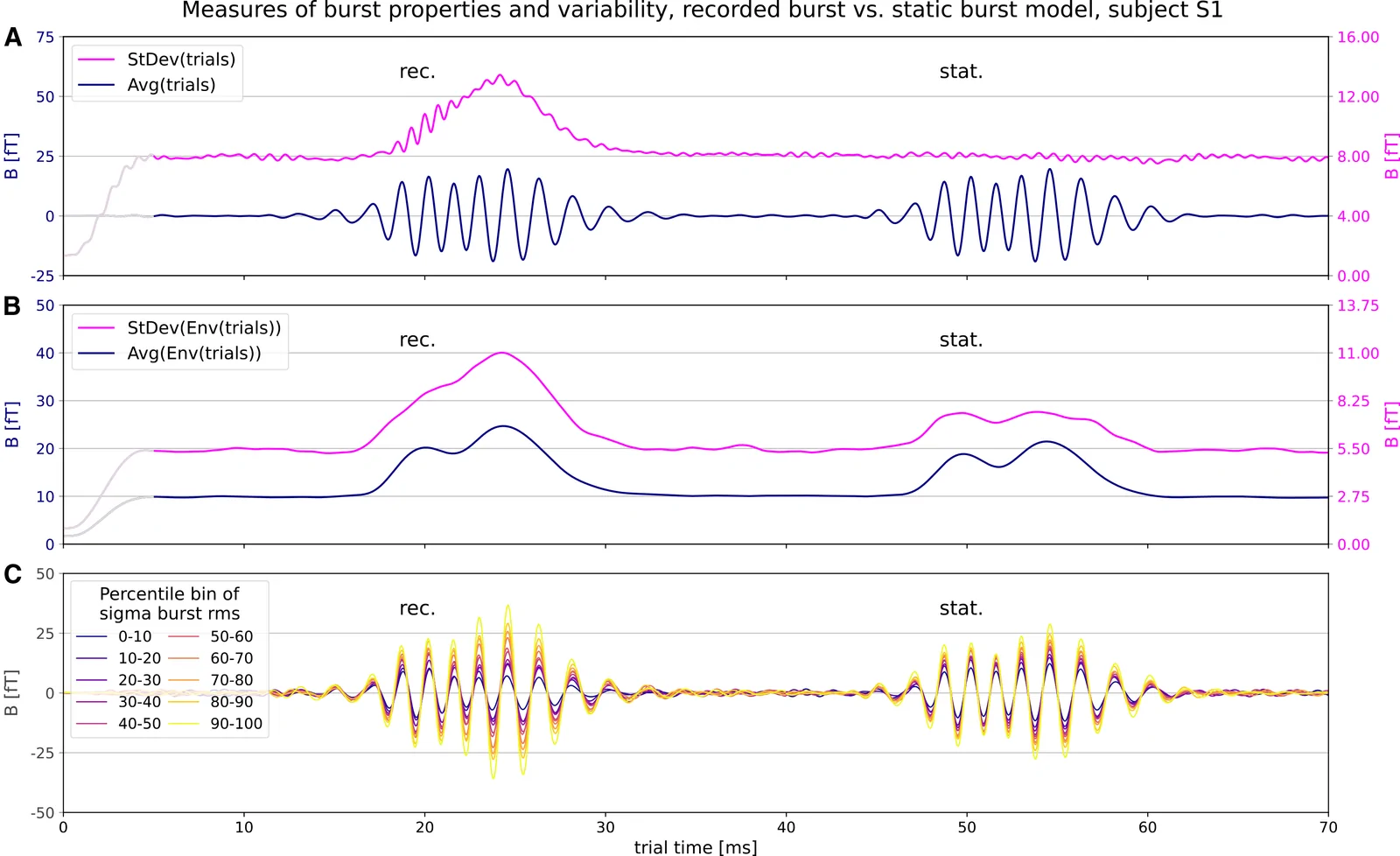
Measures used to describe properties of the burst and its variability Recorded burst (“rec.”), and static burst model (“stat.”, +30 ms relative to the recorded burst) are shown (data from subject S1). (A) Average and standard deviation of trials. (B) Average and standard deviation of the envelopes of trials. (C) Trials sorted and averaged according to their RMS percentile bin.

Critically, however, such burst variability could reflect not only the burst’s intrinsic variability, but might also reflect ongoing noise superimposed on a static, non-variable burst. Thus, an evaluation of how measures used for variability assessment are affected by ongoing noise, potentially leading to physiologically spurious results, is mandatory.

To this end, data from a previous study,^37^ augmented with additional recordings, were leveraged to investigate how ongoing noise affects the estimation of single-trial hfSER variability and to characterize the variability of the different pre- and post-synaptic burst components. Starting from a static response model, hfSER variability of increasing complexity was added to the model (cf. Figure 3 of the STAR Methods), and its parameters were fitted to match the ensemble of single-trial hfSER recordings. Eventually, this model-based analysis confirmed that hfSERs are composed of at least two functionally distinct components and that the late burst component is significantly more variable than the early one. Thus, this is a first-in-human methodological proof-of-concept that non-invasive recordings of human cortical population spiking can reveal the population spiking variability introduced by neocortical synaptic transmission.

**Figure 3:**
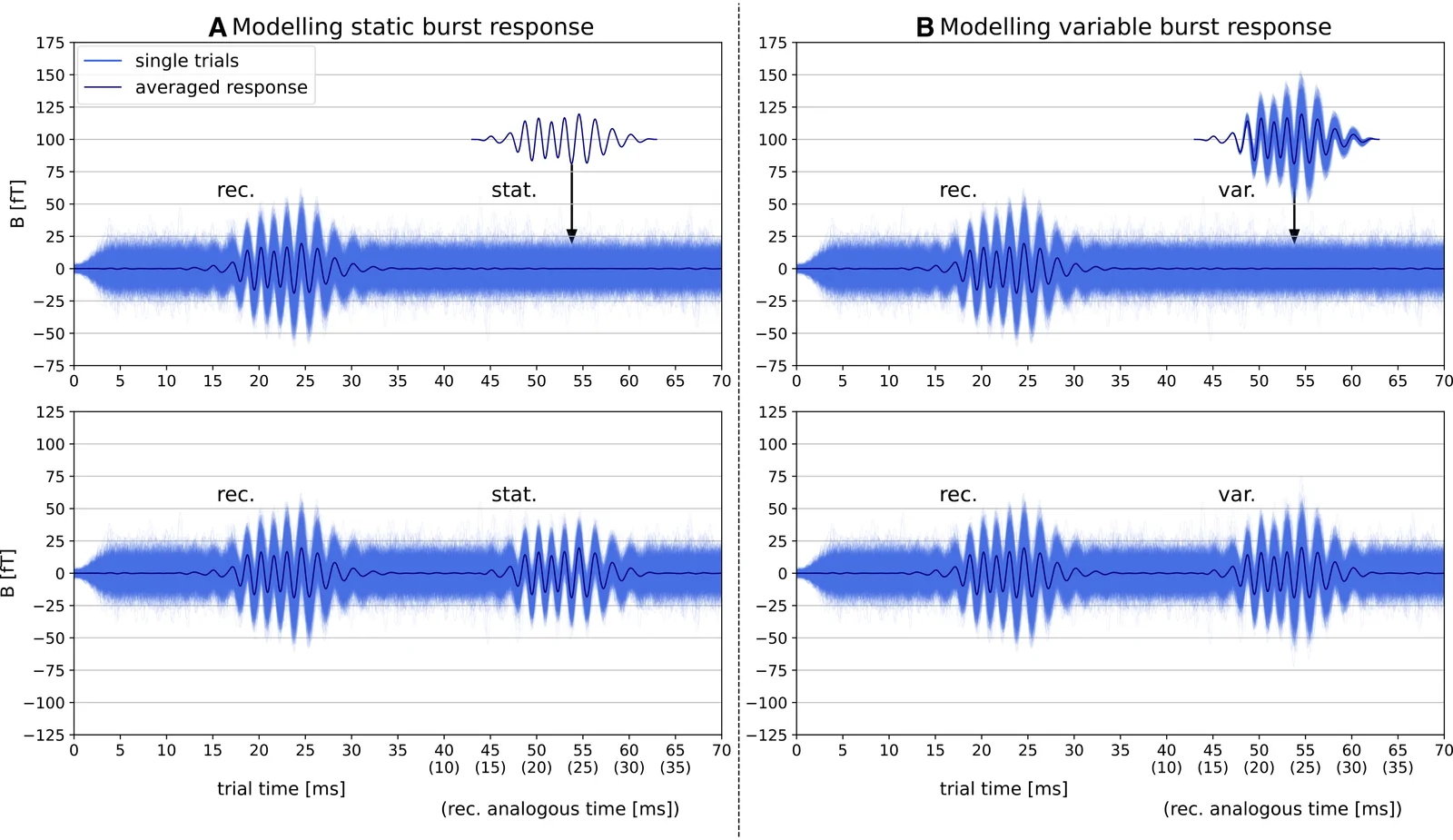
Modeling process of static and variable burst responses Modeling process of static (A) and variable burst responses (B). Single-trial (thin light-blue lines) and averaged response (thick dark-blue line) of recorded, true burst (“rec.”), static burst model (“stat.”), and variable burst model (“var.”). Exemplary data from subject S1. The upper part of the figure illustrates the superposition to generate the burst models, while the lower one shows the results. Approximately 20 ms after stimulus onset, the physiological, recorded hfSERs are observed, followed by ongoing noise. For the burst model, single-trial recordings at around 50 ms (that is, +30 ms relative to the recorded burst) were superimposed with burst responses (it was confirmed that the modeling result was not dependent on a particular selection of the noise window). In the static burst model, the ongoing recordings were superimposed with a static burst, obtained by averaging the true hfSERs, to simulate a case of response without intrinsic variability. In the variable burst model, ongoing noise was superimposed with simulated variable hfSERs (4-parameter model shown) to reproduce the observed properties of the recorded burst and get an insight into intrinsic burst variability. In the superposition process shown in the figure, only single trials took part. The presented averaged responses were calculated afterward to illustrate the variability level of single trials relative to the averaged signal.

## Results

### Recorded data and their characteristics

hfSERs were non-invasively recorded using ultralow-noise MEG (white noise level ≤0.23 fT/√Hz) of 5 healthy subjects during median nerve stimulation. A typical example of the averaged broadband and the corresponding passband-filtered (550–850 Hz) somatosensory evoked responses is shown in Figure 1. The broadband data show the low-frequency N20 response superposed with high-frequency activity, which is visible as notches and humps. These correspond one-to-one to the peaks delineated in the high-frequency passband response (sigma burst) after latency-preserving digital filtering. This excludes an origin of hfSERs as filtering artifacts, in accordance with earlier studies,^37^ and also demonstrates the effective latency-preservation of the present digital filter settings. An ensemble of single trials showing the trial-to-trial variability is shown in the Appendix, Figure P1.

A statistical model comprising a) realistic background activity, b) early pre-synaptic, and c) late post-synaptic hfSER activity was fitted to the recorded single-trial data. The variability of the hfSER components in the model was optimized to reproduce the hfSER variability of the recorded data.

### On unbiased measures of hfSER variability

To determine which measures of variability truthfully quantify intrinsic hfSER variability, the recorded hfSERs were compared to a model of the data without any variability of both pre- and post-synaptic components (model A); i.e., a static burst which resembles the average response without any single-trial variability. This serves as a litmus test for identifying potential spurious variability signals caused by burst-noise interaction.

The properties of the recorded burst and the static burst model were compared using several different measures. The average response was the same for both the recorded data (Figure 2A, “rec.”) and the static burst model (Figure 2A, “stat.”), as is expected. When the static burst was added to background activity, the standard deviation remained at the baseline noise level, consistent with no variability being added (Figure 2A, “stat.”). In contrast, the recorded bursts’ variability exceeded the noise baseline, reflecting genuine burst variability (Figure 2A, “rec.”). This is because the variance of the compound signal amounts to the sum of variances of background activity (= baseline variance) and added burst plus their doubled covariance (Equation 1). In the case of the static burst model, both the variance of the burst and its covariance with background activity are zero, so that the variance of the static burst model remains at the baseline level. Thus, compound variance and standard deviation calculated across trials are sensitive to intrinsic hfSERs’ variability, and a rise above the baseline level unequivocally demonstrates burst variability. If background activity and burst are uncorrelated—a reasonable assumption since MEG background activity >500 Hz is usually considered noise—their covariance is zero, and the compound variance’s rise above baseline directly quantifies the burst variability across trials. Accordingly, for further analyses, the standard deviation was chosen as a measure of variability, since it preserves the unit dimension of the single and averaged trials.

(Eqation 1) $$StDev\left(X+Y\right)=\sqrt{Var\left(X+Y\right)}=\sqrt{Var\left(X\right)+Var\left(Y\right)+2Cov\left(X,Y\right)}$$

StDev- standard deviation, Var- variance, Cov- covariance, X,Y- signals.

In addition, single-trial burst amplitude and its variability across trials were quantified by measures derived from the envelope of the recorded high-frequency data (Figure 2B). While the ordinary average across trials represents only phase-locked activity, the average of single-trial envelopes is phase-insensitive. Here, the average of envelopes of the recorded hfSERs (Figure 2B, “rec.”) had a larger amplitude than in the static burst model (Figure 2B, “stat.”). This indicates the presence of non-phase-locked activity in the recorded hfSERs, i.e., latency variability. The standard deviation of envelopes shows a hump for the recorded burst, but also a small one, exceeding baseline noise, is even visible for the static burst model—although there is no intrinsic variability. This is due to nonlinear properties of the envelope calculation: the envelope of the sum of two signals can be expanded as a square root of the sum of squared envelopes and a signal interaction component (Equation 2). In the static burst model, the variance of the interaction component sums up with the noise variance, which causes the visible hump. Hence, methods based on the analysis of envelope variability or rectification of the signal must be interpreted with caution.

(Equation 2) $$Env\left(X+Y\right)=\sqrt{Env{\left(X\right)}^{2}+Env{\left(Y\right)}^{2}+2\left(XY+X_{H}Y_{H}\right)}$$

*Env*-envelope of the signal, *X*,*Y* - native signals, *X*_*H*_,*Y*_*H*_ - Hilbert transformed signals.

As complementary analysis, single-trials were sorted according to the burst amplitude, quantified as root-mean-square (RMS) values, and averaged in bins of 10%-percentile width. Notably, the static burst model revealed a sorting effect, reflecting the interaction between the static burst and the ongoing noise, which can interfere constructively with the static burst, increasing its amplitude, or destructively, decreasing it. This interaction becomes visible as amplitude variability in the static burst model and leads to its sorting effect (Figure 2C, “stat.”). The sorting percentile spread, however, was much stronger for the recorded burst (Figure 2C, “rec.”), confirming the presence of the additional, intrinsic hfSER variability in the recording.

### Model-based quantification of hfSER variability

The modeling aims to explains the observed variability of the recorded burst. Clearly, the base model without added hfSER variability (model A) grossly fails to match the standard deviation across trials that was observed in the recordings (Figure 4A). Adding amplitude variability only (model B) introduced strong variability at the peaks and troughs of the burst, though, but no variability at the zero-crossings, a pattern dissimilar to the recorded data (Figure 4B). Also, adding latency variability only (model C) was not able to reproduce the smooth increase of the signal’s standard deviation throughout the entire hfSER time window, which can be observed in recorded data (Figure 4C). Only models featuring both amplitude and latency variability could approximate the general pattern of variability observed in the recordings (Figures 4D–4F). Models introducing a split into early and late burst components (Figures 4E and 4F) decreased the mean square error between model and experimental data even further. This is in agreement with previous data suggesting both amplitude and latency variability^37^^,^^38^ in hfSER recordings. Hence, only models D-E were subjected to a detailed parameter optimization, with the static burst model (model A) serving as a baseline reference.

**Figure 4:**
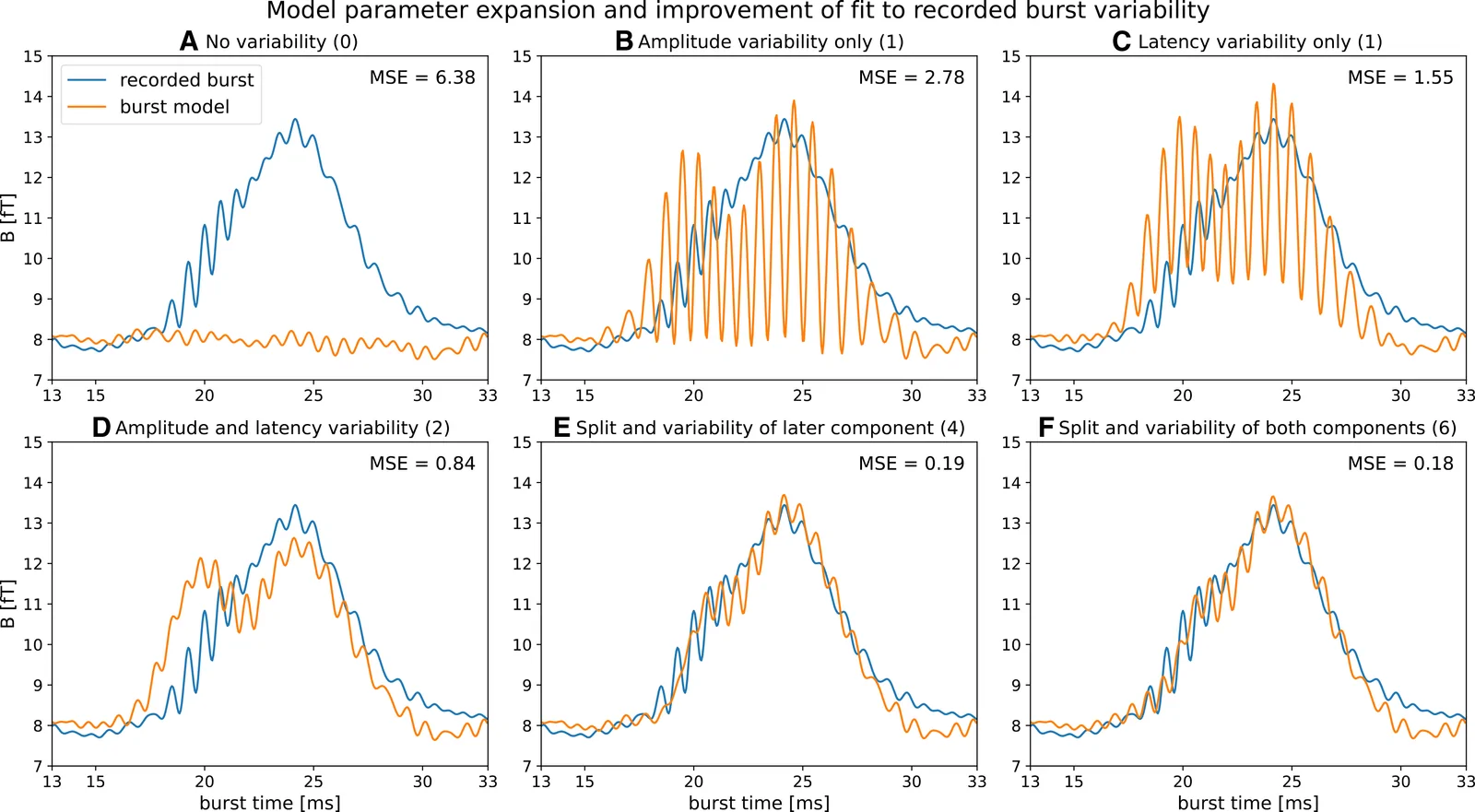
Fit between standard deviation curves of modeled and recorded bursts The number in parentheses denotes the number of optimized parameters for each model. Mean squared error (MSE) was used as a fit measure. Exemplary data from subject S1. (A) Static burst model (no intrinsic variability). (B–F) Variable burst model: (B) Amplitude variability. (C) Latency variability. (D) Amplitude and latency variability. (E) Split into early and late burst components, amplitude and latency variability of the late component. (F) Split into early and late burst components, amplitude and latency variability of both components.

Model D featured 2 parameters: an amplitude/latency variability scaler (*avs* + *lvs*), model E (4 parameters) introduced a split between early and late hfSER components with optimized division position and steepness (*div_pos* + *div_steep*) and added variability only to the late component (*lc_avs* + *lc_lvs*), and model F (6 parameters) additionally added amplitude/latency variability also to the early component of the hfSERs (*ec_avs* + *ec_lvs*). Note that adding variability to the early hfSER component only did not result in a reasonable fit (cf. Methods section).

The results of optimization (Figure 5) become gradually better with increasing number of parameters, reaching a plateau for the 4-parameter model (model E, cf. Figure 6 of the STAR Methods). To provide a proper statistical analysis, an across-subject paired-sample *t* test was applied to check if each model performed significantly better than the preceding, simpler one. The results (detailed in Appendix, Table S1) indicated that the 2-parameter model (model D) was significantly (*p* < 0.01) better than the static burst model (model A). The 4-parameter model also showed a significant (*p* < 0.01) improvement compared to the 2-parameter one. On average, the 4-parameter model explained 85% of the observable burst variability. Accordingly, this simulation provides further independent evidence for the experimentally demonstrated division into at least two functionally and neuroanatomically distinct burst components.^22^^,^^23^^,^^24^^,^^25^^,^^29^^,^^30^^,^^39^ There was no statistical evidence (*p* > 0.95) that the 6-parameter model (model F) performed better than the 4-parameter one. Notably, for the data with the highest SNR (subject S1), the 6-parameter model showed a marginally better result than the 4-parameter model.

**Figure 5:**
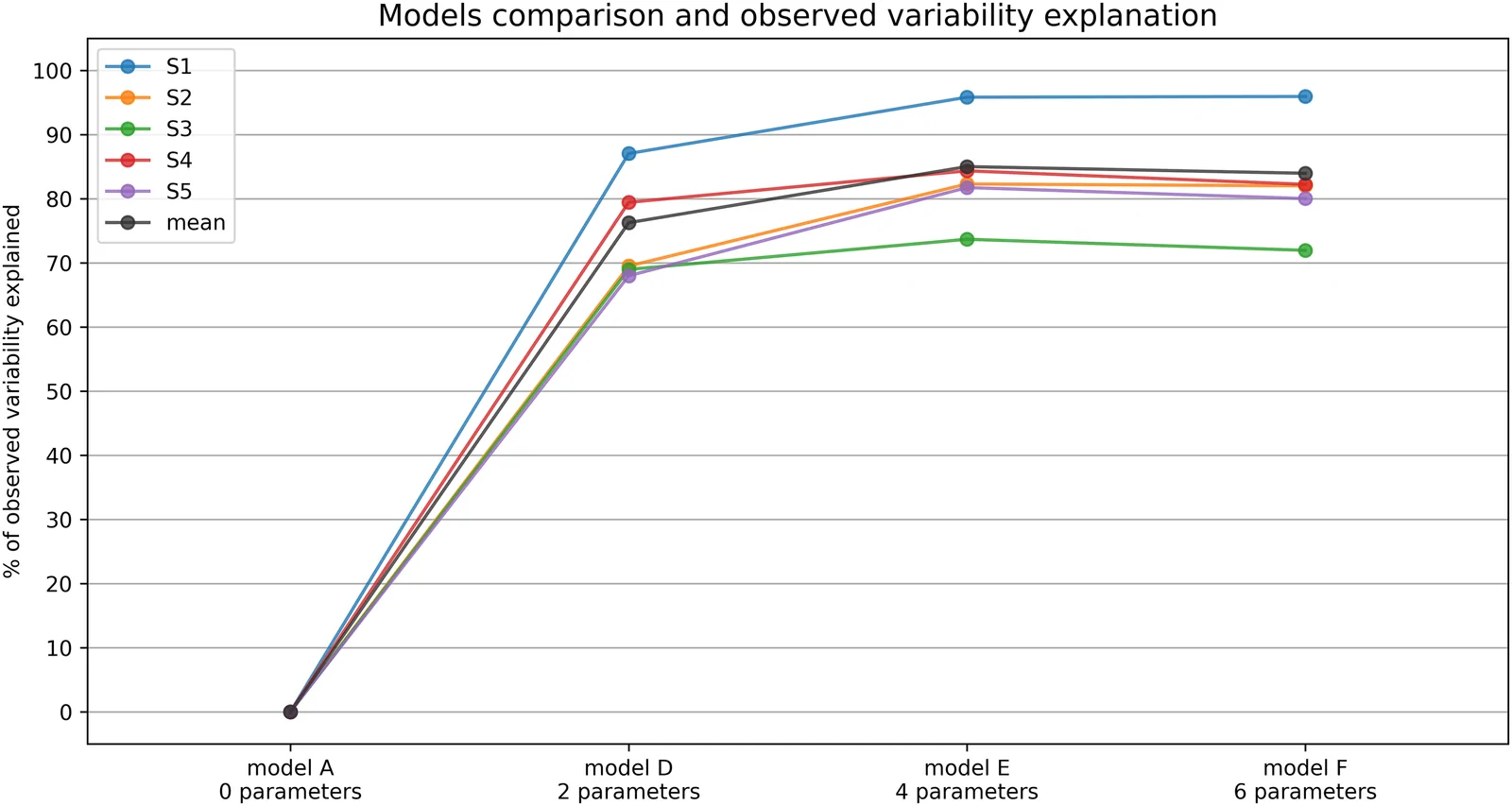
Percentage of observed burst variability explained by the model The static burst model (0-parameter, model A) was considered as a baseline reference with no variability explained (0%). A perfect fit was considered as a full explanation of the observed variability (100%). The 4-parameter model (model E) explained on average 85% of variability.

**Figure 6:**
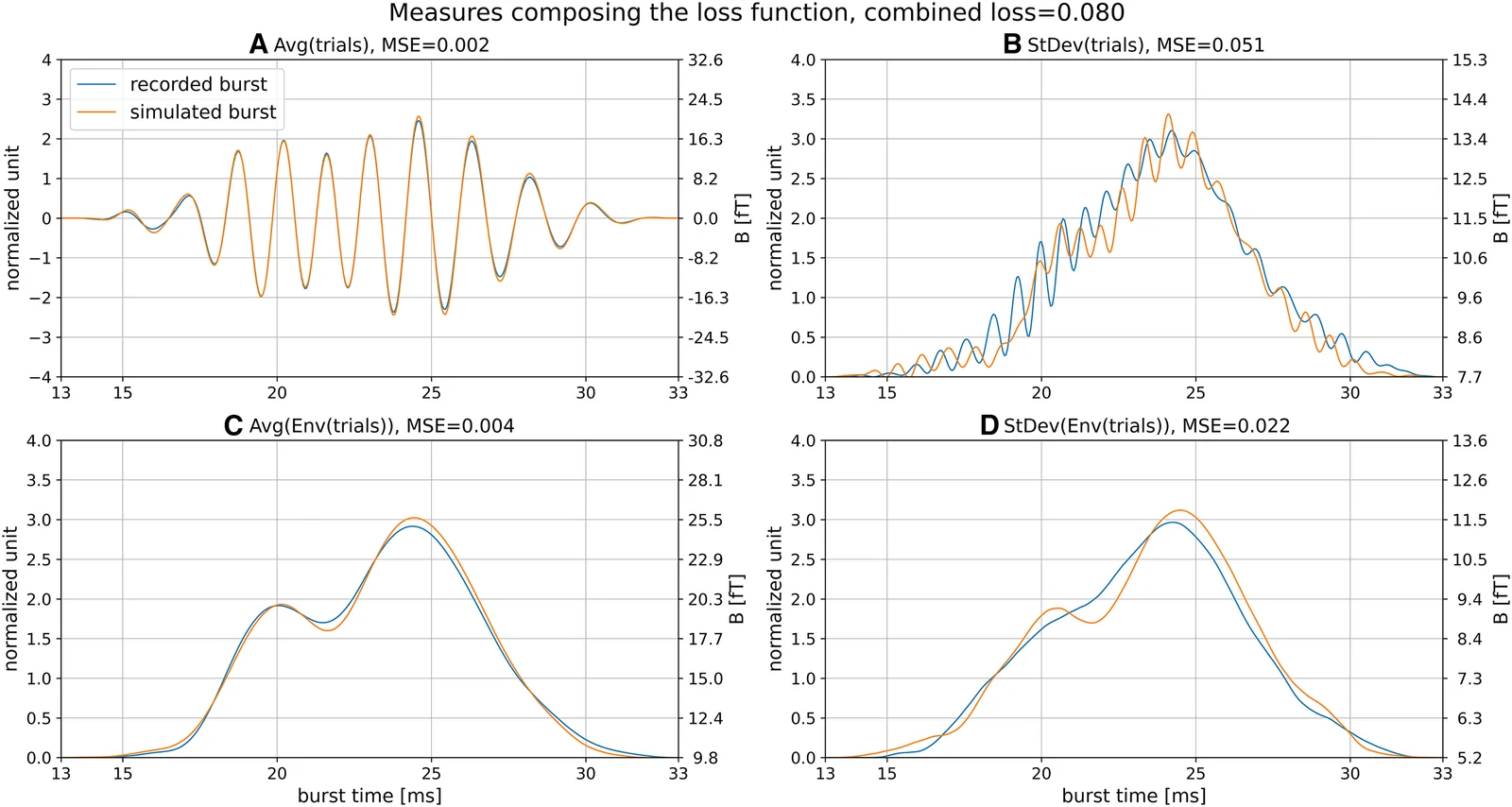
Set of measures composing the loss function (4-parameter model) For each measure, curves were normalized with respect to the recorded burst, and a Tukey window was applied to attenuate the influence at the edges of the analysis window. The mean squared error (MSE) for every pair of curves was calculated and subsequently summed to form the loss function. Exemplary data from subject S1. (A) Average of trials. (B) Standard deviation of trials. (C) Average of envelopes of trials. (D) Standard deviation of envelopes of trials.

Additionally, the optimized parameters (Appendix, Table S2) in the 6-parameter model, controlling the late component’s variability (*lc_avs*, *lc_lvs*), were significantly larger (*p* < 0.01) than those for the early component (*ec_avs*, *ec_lvs*). This reinforces the conclusion that the variability of the late component is in general sufficient to explain the observed burst variability, while expanding the model parameters to add variability to the early component also results in overfitting, given the average SNR of the data.

Accordingly, measures used to derive burst properties and variability were calculated for the 4-parameter model. Using this model, recorded hfSERs (Figure 7, “rec.”) were closely approximated by modeled hfSERs (Figure 7, “var.”). The center position of the division curve used to split the burst into early and late components was found, on average, at about one-third (0.36) of the burst length and was consistent across subjects (Appendix, Table S3). The parameters used to control the late component variability *lc_avs* and *lc_lvs* were estimated, on average, at 0.46 and 2.14 (Appendix, Table S3). The rescaling factor (*lc_acs*) to compensate for the amplitude reduction of the late component in the average hfSER in comparison to single trials, due to latency variability, amounted, on average, to 1.11 (range 1.04–1.16). Note that this is not an independent model parameter but a descriptive parameter determined within the model itself.

**Figure 7:**
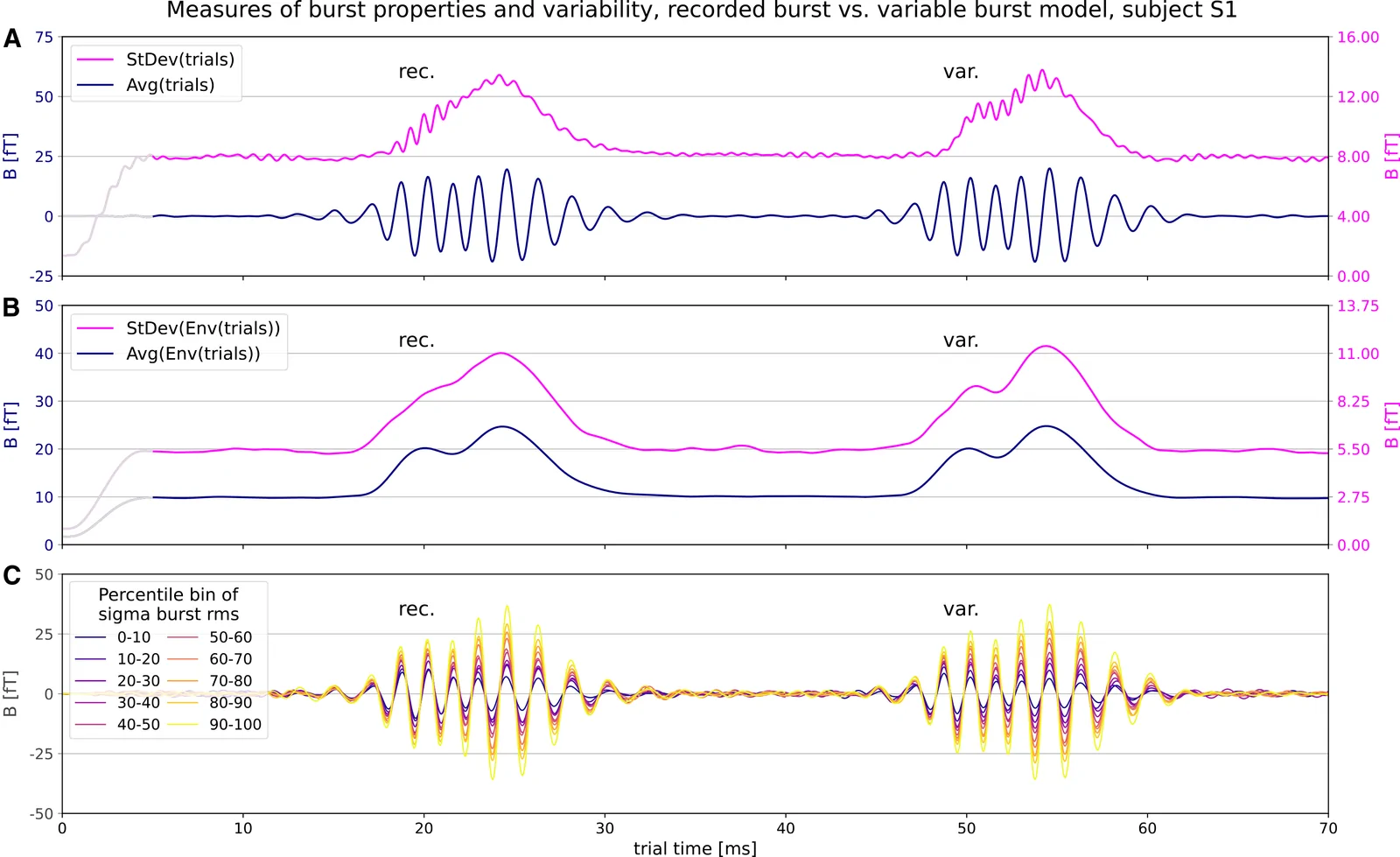
Measures used to describe properties of the burst and its variability Recorded burst (“rec.”), and fitted 4-parameter variable burst model E (“var.”, +30 ms relative to the recorded burst) are shown (data from subject S1). (A) Average and standard deviation of trials. (B) Average and standard deviation of the envelopes of trials. (C) Trials sorted and averaged according to their RMS percentile bin.

The parameters obtained during the fit procedure reflect the properties of generated amplitude and variability distributions; however, their dimension was chosen to enable best performance within the modeling pipeline. The subsequent translation of generated distributions to the physical units (ms, fT) is shown in the Appendix, Figure P2. The properties of modeled late component variability distributions (4-parameter model) for every subject are presented in Table 1.

**Table 1:** Properties of the modeled distributions for the late component variability, 4-parameter model

Subject	ER amplitude p-p [fT]	Amplitude p-p distribution mean [fT]	Amplitude p-p distribution st_dev [fT]	Latency distribution st_dev [ms]	Division curve center position [ms]
S1	38.487	44.868	19.263	0.139	19.44
S2	33.812	35.342	8.419	0.070	17.89
S3	47.535	52.121	17.064	0.102	18.77
S4	25.715	28.823	11.991	0.107	17.25
S5	41.520	47.617	25.817	0.118	18.04
mean	37.414	41.754	16.511	0.107	18.28

The division curve center position represents a post-stimulus latency in the recorded burst time window. ER - evoked response.

Complementary analyses were performed to characterize further the relation between the 4-parameter variability model and the recorded data: comparing a time-frequency representation (Figure 8), the shapes of the recorded and modeled bursts exhibited similar profiles. Only the frequency range of the modeled burst was more limited, reflecting the effect of the band-pass filter used to extract the burst from the recorded averaged data. It is worth noting that the averaged response (Figure 8A) shows an early, frequency-ascending component and a late, frequency-descending one. In contrast, the standard deviation (Figure 8B) is primarily concentrated in the late (descending) component, indicating that most of the variability occurs there. The time-frequency representation of the recorded and modeled bursts for all of the subjects is available in the Appendix, Figures TF1–TF5.

**Figure 8:**
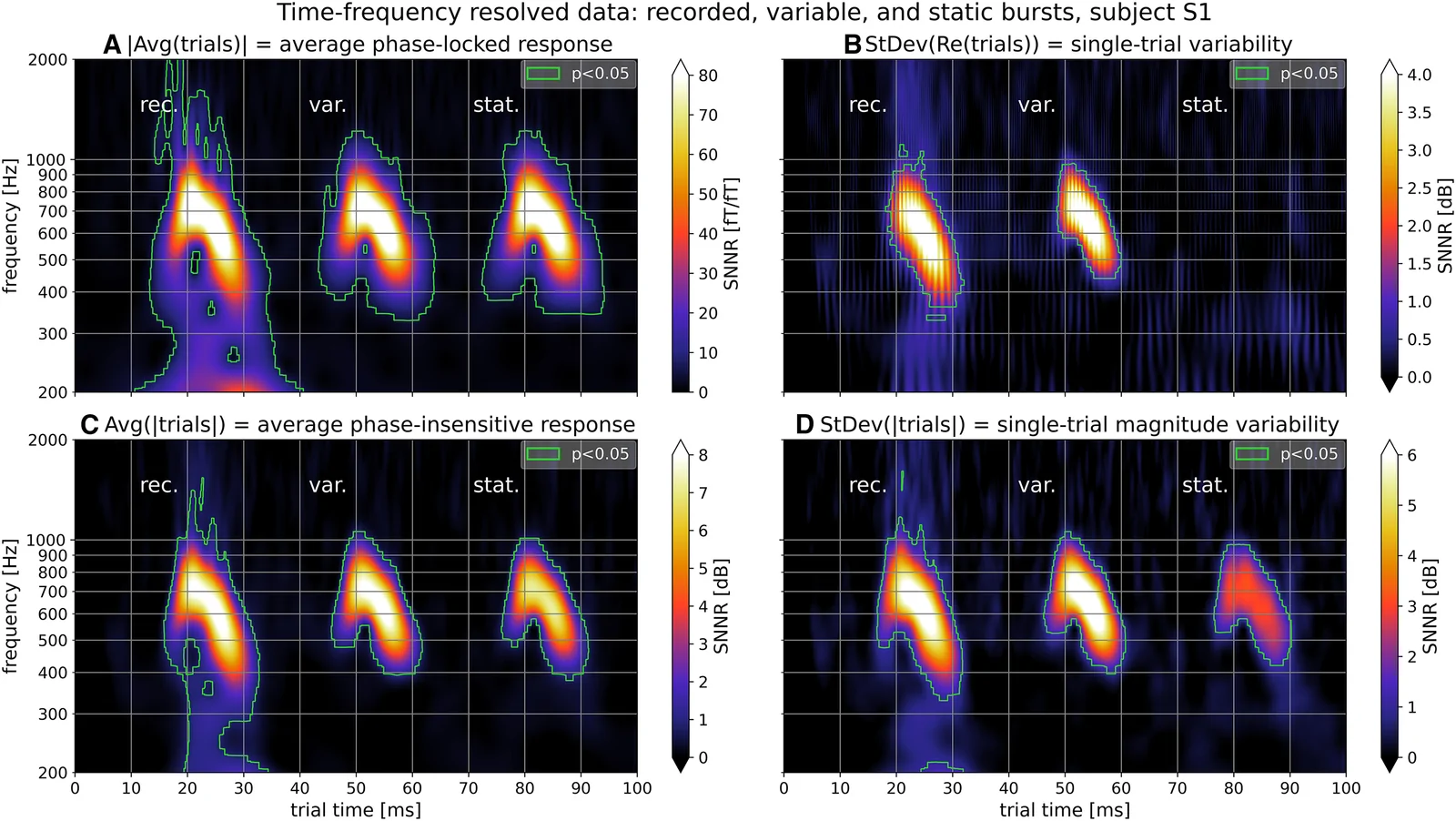
Analysis of time-frequency resolved data The modulus “| |” of complex-valued tf-data yields the magnitude, which is equivalent to the envelope calculation in time-resolved data, while “Re( )” denotes the real part of that data. Each subplot (A-D) shows recorded burst (“rec.”), 4-parameter variable burst model (“var.”, +30 ms relative to the recorded burst) and static burst model (“stat.”, +60 ms relative to the recorded burst, placed here for comparison purposes). The green encirclement indicates regions where the signal was significantly stronger than noise level (*p* < 0.05 after family-wise error correction for multiple testing). Note that only the standard deviation across real-valued single-trials (B) shows no variability in the case of simulated static burst addition and thus can faithfully represent intrinsic variability for both recorded and simulated variable bursts. Exemplary data from subject S1. (A) Average (phase-locked) response. (B) Standard deviation of the real part of single-trial data. (C) Average magnitude (phase-insensitive) response. (D) Standard deviation of single-trial magnitudes.

Stacked single-trials allow one to obtain a visual overview of both the magnitude and stationarity of the variability in the data. Figure 9 demonstrates this for the recorded burst, the 4-parameter variable burst model, and the static burst model of subject S1. A presents the real recorded data, including the burst and the subsequent ongoing noise. B shows the recorded, variable, and static bursts after superposition on the ongoing noise. C shows the burst models before superposition with single trials, showing the burst response appearance without the influence of noise. The recorded and simulated bursts in B show trial-to-trial variability, with some peaks missing and others with higher amplitude. Comparing the simulated bursts (B) to the ongoing noise (A) and to the bursts without noise (C) reveals that part of this variability is caused by burst-noise interactions. For example, the larger amplitudes observed in the group of simulated variable bursts between trials 650 and 660 result from constructive interference with the noise.

**Figure 9:**
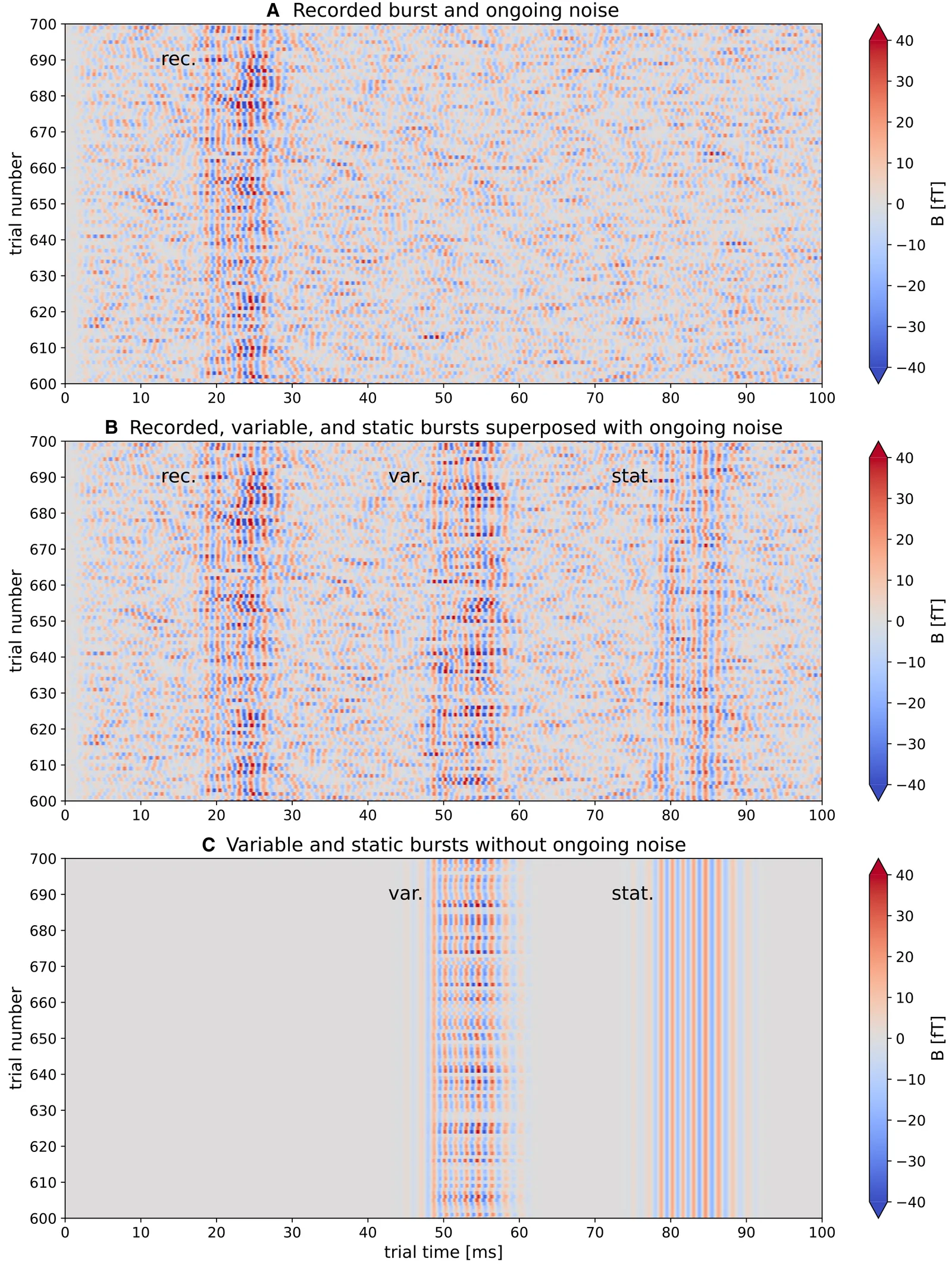
Single-trials stacked on each other (data from subject S1) (A) Recorded burst (“rec.”) and ongoing noise. (B) Recorded burst (“rec.”), 4-parameter variable burst model (“var.”), and static burst model (“stat.”) superposed on ongoing noise. (C) Variable (“var.”) and static (“stat.”) bursts without ongoing noise. Variable burst model (“var.”) is located +30 ms relative to the recorded burst and static burst model (“stat.) +60 ms, placed here for comparison purposes.

## Discussion

The advances obtained from this study are 3-fold: (1) Analysis of the static burst model reveals that the calculation of standard deviation of activity across trials is a reliable marker of genuine hfSER variability, whereas measures based on the power or envelope of single-trial data are susceptible to spurious detection of hfSER variability, even in the absence of it. This, importantly, also affects calculating the variability of hfSER magnitude in time-frequency representations as performed earlier.^38^ (2) The development of the model-based analysis provides interpretable results by parametrizing the variability into amplitude and latency variability, additionally allowing different variability parameters for the early and late hfSER components. (3) The analysis proves the concept that non-invasive low-noise measurements of cortical single-trial population spike responses can be analyzed at such detail as to quantify the differential variability of individual sequential burst components, eventually revealing the variability of human neocortical synaptic transmission.

This interpretation is strongly supported by previous physiological and anatomical studies suggesting that the burst is indeed comprised of at least two components: an early, pre-synaptic one, originating from thalamo-cortical fibers, and a late, post-synaptic one, caused by intracortical neural activity.^16^^,^^22^ The post-synaptic origin of the late burst component was demonstrated by the intracortical injection of the broad-spectrum synaptic blocker kynurenic acid, which almost completely abolished the 2nd half of the burst response evoked by snout stimulation in the pig while retaining the first half of the burst.^30^ In the present study, a mixed peripheral nerve was stimulated above motor threshold to maximize the amplitude of the responses, which is the recommended procedure by the International Federation of Clinical Neurophysiology for clinical SER measurements.^40^ The effect of such suprathreshold median nerve stimulation on early cortical SERs has, however, been shown to be small,^41^^,^^42^ and an expected prolongation of the burst due to late-arriving muscle spindle re-afferents during stimulation above motor threshold cannot be observed practically.^43^ Thus, muscle-spindle reafferents are not likely to contribute significantly to the hfSER burst modeled here.

While backed up by anatomy and previous recordings, this model is—just as any model—a simplification of the actual hfSER physiology. In reality, the observable activity represents the superposed activity of neuronal ensembles with distributed location and statistical neuronal spiking properties. Non-invasive measurements with macroelectrodes cannot fully disentangle this multiplicity of neuronal activity at the source level. Accordingly, the model does not include synaptic transmission as its own model parameter. The emergence of increased variability in the 2nd half of the macroscopic burst response, however, suggests an additional processing step preceding this part, and the latency-jitter introduced to the 2nd burst component (0.1–0.13 ms) is consistent with the monosynaptic transmission^44^ between thalamocortical afferents and Area 3b neurons.^45^^,^^46^^,^^47^ Notably, this interpretation was robust to constraining the steepness of the division curve between the first and 2nd half of the burst, which allowed for a greater overlap between both components (data not shown).

Derived from the amplitude of the average hfSER at the scalp surface, the size of this ensemble had been estimated as approx. 1200.^37^ The results of the present study, however, indicate that the amplitude of the average hfSER underestimates the true single-trial hfSER amplitude due to the presence of inter-trial latency variability. For the 4-parameter model (model E) the single-trial burst amplitude had to be up-scaled by an average factor of 1.11 to compensate for the latency variability. Accordingly, the true number of neurons contributing to the hfSER response needs to be larger than the previous estimate by this factor, yielding approx. 1300 neurons.

Additionally, given the possibility of asynchronous spiking within the neuronal ensemble generating the macroscopic hfSER,^48^ also this estimate only provides a lower bound under the assumption of perfect ensemble synchrony. Precise estimates would require invasive multi-unit recordings to estimate the degree of ensemble synchrony during hfSER generation.

Moreover, the observed burst was modeled as a superposition of maximally two components. The complex spatial topography of the hfSER in a frequency range extending to >1000 Hz suggests the presence of even further hfSER components,^33^^,^^39^^,^^49^ and disentangling multiple sources and the interaction between them would require a further improvement of SNR.

Generally, the SNR dictates the interpretability of experimental data, with higher SNR potentially revealing results that had previously been hidden under noise. This is paradigmatically demonstrated by the progress made in the analysis of non-invasive human hfSER records in the past decades, achieved in parallel with concurrent advances of recording and analysis methods—from a mere notion of the average hfSER occurrence,^17^^,^^50^ to hfSER source analysis,^16^^,^^22^^,^^39^ hfSER single-trial detection,^36^ description of single-trial hfSER variability,^37^^,^^38^ up to the detailed analysis of single-trial variability on the component level described in this study. For this reason, the solution obtained in the present study should also be regarded as a preliminary approximation, and this may account for why “only” 85% of variability is explained by the final model. A possible indication for a next insight, currently hidden under noise, is the finding that for the subject with the highest SNR (subject S1), the 6-parameter model performed better than the 4-parameter model. This indicates a possible source of variability even in the pre-synaptic component, and future research can be directed to disentangle whether this is due to input variability at the stimulus level or whether this variability is introduced at the level of the peripheral nerve, spine,^51^ or thalamus.^28^

A limitation to the interpretation of the findings is the number of subjects used to record ultralow-noise MEG in this study. Therefore, while consistent in this set of subjects, the physiologic results are not generalizable, but, critically, the study proves for the first time the concept that non-invasive ultralow-noise MEG can achieve the signal-to-noise ratio of hfSERs required to demonstrate the variability introduced by synaptic transmission in the human neocortex. This further corroborates the notion that non-invasive recordings of high-frequency neural data (>500 Hz) provide a robust way to analyze cortical population spiking in healthy subjects. This approach might therefore aid in confirming findings inferred from recordings with sharp microelectrodes during surgery in the human brain of patients with epilepsy, movement disorders, and psychiatric diseases.^8^^,^^10^^,^^11^^,^^13^^,^^52^

Moreover, high-frequency brain activity has also sparked interest in the diagnosis of brain diseases such as epilepsy^53^^,^^54^^,^^55^ and Parkinson’s disease.^56^^,^^57^^,^^58^ Especially in epilepsy, high-frequency oscillations (HFOs) were shown to be more specific biomarkers of the disease than the macroscopic interictal discharges commonly denoted as “spikes”.^53^^,^^59^ Epileptic HFOs are commonly divided into ripple (80–250 Hz) and fast ripple activity (>250 Hz),^60^ the latter of which has the highest specificity for the seizure onset zone^61^ and, similar to the hfSER characterized here, was shown to be a correlate of cortical population spiking activity.^62^^,^^63^

Fast ripples can be detected non-invasively using MEG,^64^ but their clinical utility appears limited, as they can be observed only in a minority of patients.^59^ While this holds when using standard recording equipment, simulation studies^65^ demonstrated that it is not the signal amplitude alone but the SNR that governs whether or not HFOs can be detected at the scalp level. The reliably triggered occurrence of hfSERs makes them an ideal “workhorse paradigm” to maximize the SNR in this frequency range, and basic research on hfSERs can hereby also inspire studies on epileptic HFOs. For example, spatial filtering techniques, successful in increasing the SNR of hfSERs,^31^ were also implemented to detect epileptic HFOs.^66^ Similarly, the use of low-noise EEG equipment, once constructed to detect hfSERs non-invasively, allowed to record fast ripples at more than twice the frequency during intraoperative electrocorticography when compared to concurrent standard recordings in patients with epilepsy.^67^

To promote the use of ultralow-noise MEG, the detailed steps to reduce the MEG noise level were made public,^35^^,^^68^ and the device was specifically constructed to be operated in a widely available moderately shielded room.

In the future, results that are currently obtainable only when using invasive measurements might be attainable using ultralow-noise high-frequency MEG, reducing the procedural risk for the patients and permitting novel electrophysiologic diagnostics. Interestingly, high-frequency bursting is a physiological hallmark of the healthy hippocampus,^69^^,^^70^^,^^71^ and the opportunity to measure changes in hippocampal HFO activity non-invasively might provide new electrophysiologic markers for the diagnosis and monitoring of memory disorders such as Alzheimer’s disease.^72^

Remarkably, the possibility to characterize the variability of neuromagnetic cortical population spiking was provided by only a single MEG channel. Considering this just as a starting point, the development of a multi-channel device with comparable noise performance has recently been completed^73^ and will allow for the analysis of the spatial distribution of burst components and the application of spatial filters to increase the signal-to-noise ratio, potentially offering a clearer view of the variability of the individual burst components. This will enable tracking the evolution of hfSER variability over time. A possible application is to investigate whether cortical population spiking is related to concurrent regional MEG rhythms (e.g., alpha or theta/delta phase and amplitude) and physiologic parameters such as attention and drowsiness, or changing independently. Additionally, it has been shown that hfSERs follow a somatotopic arrangement,^74^ and this multi-channel system will make it possible to determine whether variability of cortical population spiking in the healthy human brain is topographically independent or correlated across the somatosensory cortex. Besides the study of the healthy human brain, this will also increase the possibility to measure regional changes of cortical excitability, which is of utmost interest, e.g., in the diagnostic procedures of epilepsies.^75^

## Limitations of study

The present study was designed as a proof of concept. Accordingly, its main limitation is the restricted generalizability of its findings to the broader population (data were collected from 5 subjects). Additionally, the interpretation of its findings relies on previous research, and the variability of synaptic transmission was not measured directly (i.e., invasively) but inferred from modeling of non-invasive MEG data. The modeling approach was designed to assess evoked activity and is not directly suitable for spontaneous activity. The ultralow-noise MEG system had a residual noise level of ≤0.23 fT/√Hz, which was taken into account in the model. The data were obtained with a single-channel sensor; in future studies, a recently developed multichannel MEG system will be employed, enabling spatial filtering and multi-source analysis. While the non-commercial ultralow-noise MEG system used here is presently available only at PTB Berlin, its design principles have been published.

## Resource availability

### Lead contact

Requests for further information and resources should be directed to and will be fulfilled by the lead contact, Lukasz Radzinski (lukasz.radzinski@gmail.com).

### Materials availability

This study did not generate new unique reagents.

### Data and code availability

• Raw single-trial MEG data reported in this study cannot be deposited in a public repository because the informed consent obtained from participants does not cover unrestricted public sharing of individual neurophysiological data. To request access, contact the lead contact. Data will be shared under a Data Transfer Agreement that specifies data ownership and citation requirements and restricts use to non-commercial research; it does not limit the ability of researchers to reproduce the reported analyses or to perform new analyses. In addition, spectra of the data, trial-averaged hfSER waveforms, complete results of every modeling run, and the final fitted model parameters for all subjects have been deposited at Zenodo: https://doi.org/10.5281/zenodo.22547578 and are publicly available as of the date of publication.
• All original code has been deposited together with the summary statistics of the data at Zenodo: https://doi.org/10.5281/zenodo.22547578 and is publicly available as of the date of publication.
• Any additional information required to reanalyze the data reported in this paper is available from the lead contact upon request.

## Acknowledgments

This study was supported by the 10.13039/501100001659Deutsche Forschungsgemeinschaft (10.13039/501100001659German Research Foundation) Project ID 511192033.

## Author contributions

Conceptualization: L.R., G.W., and G.C.; manuscript writing and editing: L.R.; primary reviewers: G.W. and G.C.; contributing reviewers: B.B., R.K., J.B., and S.A.; data analysis: L.R., G.W., G.C., and B.B.; data collection: G.W., G.C., R.K., J.B., S.A., and L.R.; project administration: G.W. and R.K.

## Declaration of interests

The authors declare no competing interest.

## STAR★Methods

### Key resources table

**Table:** 

REAGENT or RESOURCE	SOURCE	IDENTIFIER
**Software and algorithms**		

Python v3.12.12	Python	https://www.python.org/
NumPy v1.26.0	NumPy^76^	https://numpy.org/
SciPy v1.15.3	SciPy^77^	https://scipy.org/
TensorFlow v2.17.0	TensorFlow^78^^,^^79^	https://www.tensorflow.org/
Matplotlib v3.8.0	Matplotlib^80^	https://matplotlib.org/
meet v2024.05.06	Custom-developed^31^^,^^36^^,^^81^	https://github.com/neurophysics/meet
BurstModeling	This paper	https://doi.org/10.5281/zenodo.22547578

**Other**		

MEG data	This paper	N/A

### Experimental model and study participant details

The data were analyzed from 5 healthy participants (30–60 years; 4 male, 1 female), denoted as subjects S1-S5. All subjects provided informed consent to participate. The study protocol was approved by the Ethics Committee of the PTB (decision ID PTB2022-2). Analyses by age and sex were not performed, as these variables lay outside the predefined scope and objectives of this proof-of-concept study.

### Method details

#### Data acquisition and preprocessing

The measurements were performed using an ultralow-noise one-channel SQUID-based MEG system. The system was custom-made and developed at the Physikalisch-Technische Bundesanstalt (PTB) for recording cerebral low-amplitude high-frequency activity.^37^ The raw data for subjects S1-S4 were re-analyzed, and a detailed description of these data is provided in an earlier report about single-trial hfSER detection.^37^ In these data, the MEG device provided for a white noise level of 0.18 fT/√Hz. Data for S5 were newly recorded using a different SQUID sensor providing a white noise level of 0.23 fT/√Hz.

During the recording, each subject was lying on the left side, the head stabilized by a vacuum cushion, and the MEG sensor was placed over the right parietal region of the head near the primary somatosensory cortex. The median nerve at the left wrist was stimulated at a frequency of 3.27 Hz with an intensity above motor threshold, with subjects S1-S4 stimulated for 10 min (∼2000 trials) and S5 for 5 min (∼1000 trials). The recording was digitised at a 20 kHz sampling rate.

The data were preprocessed to eliminate artifacts as the stimulation impulse caused a spike-like artifact in the data, which was removed by monotone cubic Hermite interpolation,^82^ with an interpolation time window of 6 ms centered at the artifact. To extract hfSERs, the signal was band-pass filtered using a 303-tap FIR filter applied in forward and backward direction to achieve zero time delay. Using a time-frequency display of the measured average hfSER (S-transform),^83^ the passband was adjusted individually for each subject to optimally contain the single-trial hfSERs, leading to a passband of 500–800 Hz for subjects S1, S2 and 550–850 Hz for subjects S3-S5, (Appendix, Table S4). The continuous signal was segmented into trials (duration = interstimulus interval), with t = 0 ms denoting the stimulus onset. Signal quality in some of the trials suffered from increased surface electromyographic (EMG) activity or technical noise, which resulted in elevated average amplitude of the trial. RMS (root-mean-square ) amplitude was used as a measure of average amplitude and calculated over the whole period of every trial. Trials with RMS above Q3 (third quartile) + 3 × IQR (inter-quartile range) were classified by Tukey’s fence as far outliers and rejected together with trials adjacent to them.^84^

#### Derivation of the burst variability model

Previous analyses^37^^,^^38^ demonstrated the presence of hfSER-variability in low-noise recordings. Additionally, this variability was shown to be independent of concurrent low-frequency responses^37^ and may thus reflect instantaneous state changes of the neuronal networks generating these high-frequency responses. The goal of the present analysis is to establish a simulation model approaching the hfSER variability in the recorded data of every subject and, thereby, to quantify and analyze the variability of these macroscopic spike-like responses.

In order to model the variability of hfSERs, a burst template was extracted from the recorded data of every subject (Figure 3, “rec.”) by obtaining the average hfSER within an individually adjusted time window (Appendix, Table S4). Adding this burst template to background activity in a late post-stimulus period devoid of evoked activity yielded the model of a static burst added to ongoing noise (Figure 3, “stat.”). For the variable burst model, random variability was added to this static template to emulate variable single-trials, which were then added to the ongoing noise (Figure 3, “var.”).

The ‘types’ of single-trial alterations to introduce hfSER variability can be conceptualized as scaling (amplitude variability), shifting (latency variability), and combinations thereof. Additionally, these alterations can be applied to the entire burst as a whole or to some extracted components of it. Here, the complexity of the model was increased stepwise to eventually enable it to match the recorded burst variability.

The following models of variability were tested.

A. no variability, which is the static burst model (Figure 3, “stat.”) and serves as a baseline to compare the other models to (0 optimized parameters).
B. amplitude variability: for each trial, the static burst response was multiplied with a random scaling factor following a Gaussian distribution. The distribution was centered around 1 and its width was controlled by an *amplitude variability scaler* (*avs*) parameter. The softplus function (Equation 3)^85^ was applied to the distribution to prevent the amplitude from falling below 0, which would mean a flip in the polarity of the oscillation. (1 optimized parameter: *avs*).

(Equation 3) $$softplus\left(x\right)=\frac{1}{\beta }\ln\left(1+e^{\beta x}\right)$$

β - function sharpness parameter, β=5.

C. latency variability, introduced by Gaussian-distributed random shifts of each trial along the time axis to the left or right: the distribution was centered around 0 and its width was controlled by a *latency variability scaler* (*lvs*) parameter. Since latency variability leads to a decrease of the amplitude of the average hfSER by reducing the phase consistency across trials, the single-trial amplitudes were subsequently rescaled by a single multiplication factor - *amplitude compensation scaler* (*acs*) - scaled to match the amplitude of the average hfSER. (1 optimized parameter: *lvs*).
D. amplitude and latency variabilities combined, which were controlled by two independent parameters. (2 optimized parameters: *avs*, *lvs*).
E. burst split into two components and variability applied to the late component only: hfSER responses were shown earlier to consist of at least two components, with evidence converging from different sensitivity to stimulation parameters,^25^^,^^29^ different topographies,^22^^,^^39^ and selective pharmacologic sensitivity^24^^,^^30^ of the burst components. For the split, a sigmoid curve was used (Equation 4, Appendix, Figure P3), controlled by two parameters, one for its steepness, *div_steep*, and one for its center position, *div_pos*, which was expressed as a ratio of the total burst length. The amplitude and latency variability was subsequently imposed on the late component, as this part of the recorded burst appeared to show the largest variability. The late component amplitude and latency variability were controlled by a *late component amplitude variability scaler* (*lc_avs*) and a *late component latency variability scaler* (*lc_lvs*), correspondingly. The decrease of the evoked response amplitude caused by the late component latency variability was compensated by the internally calculated *late component amplitude compensation scaler* (*lc_acs*). (4 optimized parameters: *div_steep*, *div_pos*, *lc_avs*, *lc_lvs*).

(Equation 4) $$sigmoid\left(x\right)=\frac{1}{1+e^{-x}},x=\mathrm{div}\_\text{steep}\cdot \left(t_{ms}-{\mathrm{div}\_\text{pos}}_{ms}\right)$$

$${\mathrm{div}\_\text{pos}}_{ms}=\mathrm{div}\_\text{pos}\cdot \left({\text{burst}\_\text{wnd}}_{end}-{\text{burst}\_\text{wnd}}_{start}\right)+{\text{burst}\_\text{wnd}}_{start}$$

$${burst}_{wnd}-\text{burst}\;\text{time}\;\text{window}$$

F. burst split into two components and variability of both components: variability of amplitude and latency was added to both components, independently for each with *ec_avs/ec_lvs* denoting the parameters for an early component amplitude*/*latency variability scaler and *ec_acs* for early component amplitude compensation. (6 optimized parameters: *div_steep*, *div_pos*, *ec_avs*, *ec_lvs*, *lc_avs*, *lc_lvs*)

The amplitude variability scalers (*avs*, *ec_avs*, *lc_avs*) correspond to the standard deviation of modeled amplitude Gaussian distribution, while the amplitude compensation scalers (*acs*, *ec_acs*, *lc_acs*) to its mean. They are expressed as multiplicative units to scale the amplitude of the measured evoked response. The latency variability scalers (*lvs*, *ec_lvs*, *lc_lvs*) correspond to the standard deviation of modeled latency Gaussian distribution, which is zero-centered around the latency of the subject’s evoked response. They are expressed in time samples (0.05 ms).

It should be noted that the theoretically possible model “two components, added variability to the *early* component” could not be justified in practice, since the optimization of this model typically led to an ‘early’ component spanning nearly the entire hfSER time window, effectively resulting in a model similar to one without a split.

The flowchart of the entire burst modeling process is shown in the Appendix, Figure P4.

#### Optimization and validation of the model

Modeling the burst properties requires defining a relevant loss function. To optimally match the burst model with the characteristics of the recorded burst, four signal representations, calculated in the high-frequency passband and the selected hfSER time window, were taken into account: the averaged response (Figure 6A), the standard deviation across trials (Figure 6B), the average of single-trial envelopes (Figure 6C), quantifying single-trial power, and the standard deviation of the single-trial envelopes across trials (Figure 6D). The envelope of each trial was calculated as modulus of the analytic signal, consisting of the real-valued and Hilbert transformed (imaginary-valued) data, according to Equation 5.^86^^,^^87^ All measures were calculated for the time window of the recorded and simulated bursts. In order to balance the influence of each measure on the final loss and to improve comparability across measures and subjects, the modeled and recorded measures were shifted onto a zero-baseline (to provide quantification only of the excess over baseline noise) and normalized such that each recorded measure had a variance of 1. After normalization, a Tukey window (α = 0.4)^88^ was applied to the measures, to attenuate the influence at the edges of the burst window. Subsequently, the mean squared errors between each modeled and recorded measure were calculated, and all four errors were ultimately summed to serve as final loss.

(Equation 5) $$Env\left(X\right)=\sqrt{X^{2}+X_{H}^{2}}$$

Env - envelope of the signal, X - real-valued signal, $X_{H}$ - Hilbert transformed signal.

The goal of the subsequent optimization was to find the parameter set for which the loss function reaches the global minimum in its solution hyperspace. To do so, a variant of quasi-Newtonian method (L-BFGS-B algorithm)^89^ with dual annealing^90^ was used. Since individual runs could get trapped in local minima, five independent runs were performed for each optimization, with each run starting at a different, randomly chosen point in the hyperspace which increases the chance to eventually find the globally best solution. Most runs in every optimization found the same set of parameters, presumably indicating the global minimum of the loss function (Appendix, Figure P5).

To ensure the validity of the results obtained for the different numbers of optimized parameters, training and testing of the model was performed on two independent datasets. Therefore, trials from each subject were randomly split into 2 equal subsets 5 times. In every split, one subset was at first used for training and the other for testing, and then their roles were reversed, resulting in 10 outcomes per subject in total, which were averaged in the end. Although the recording for subject S5 was shorter than for the others, the same number of simulated single-trials as in the other subjects was generated, re-utilizing the recorded high-frequency background activity +30 ms after the recorded hfSERs, to avoid undersampling the random distributions used to model the statistical properties of added variability in this simulation.

#### Software and packages

Data were processed using the Python programming language (v3.12.12), employing scientific packages for data analysis and visualization, i.e., NumPy (v1.26.0),^76^ SciPy (v1.15.3),^77^ TensorFlow (v2.17.0),^78^^,^^79^ Matplotlib (v3.8.0),^80^ and a custom-developed package, ‘meet’ (v2024.05.06, https://github.com/neurophysics/meet).^31^^,^^36^^,^^81^

### Quantification and statistical analysis

The model results were compared and statistically analyzed. Models of increasing complexity were evaluated using a one-sided paired-sample *t* test, comparing performance across subjects. The analysis assessed whether models with a higher number of parameters performed significantly better.

To assess the statistical significance of recorded and modeled burst responses against noise level, data were time-frequency transformed using the S transform.^83^ Subsequently, a *t* test for the difference of means was conducted for each time-frequency bin against the time-frequency bins in a noise-only period, devoid of evoked activity (100–200 ms) with variance estimated by a Jackknife procedure. Family-wise error corrected *p*-values were obtained using a bootstrap test (*N* = 200 iterations).^91^

The results of the statistical analysis and calculated parameters are reported in model-based quantification of hfSER variability.
