# Supplementary material for: Variance of human cortical synaptic transmission inferred from non-invasive magnetoencephalography of somatosensory evoked population spikes
Source: iScience. 2026 Oct 20;29(11):117880. doi: 10.1016/j.isci.2026.117880 (PMC13717894; doi:10.1016/j.isci.2026.117880)
Supplement: Supplementary file 1 [file mmc1.pdf]

iScience, Volume *29*

Supplemental information

Variance of human cortical synaptic transmission inferred from non-invasive magnetoencephalography of somatosensory evoked population spikes

Lukasz Radzinski, Rainer Körber, Jim Barnes, Soudabeh Arsalani, Benjamin Blankertz, Gabriel Curio, and Gunnar Waterstraat

APPENDIX

The appendix contains additional figures and tables that support the main manuscript. Figure P1 shows the ensemble of single trials. Figure P2 presents the modelled variability distribution for the late component. Figure P3 shows the extraction process of early and late burst components. Figures P4 and P5 are related to the modelling process and show the flowchart of the modelling and training loss curves, respectively. Table 1 presents the result for models across subjects, and Tables 2 and 3 final values of optimized parameters for the 6-parameter and 4-parameter models, correspondingly. Table 4 contains information about frequency band and time window used for hfSERs extraction in all subjects. Figures TF1-TF5 present the time-frequency resolved data of the recorded and modelled bursts for subjects S1-S5.

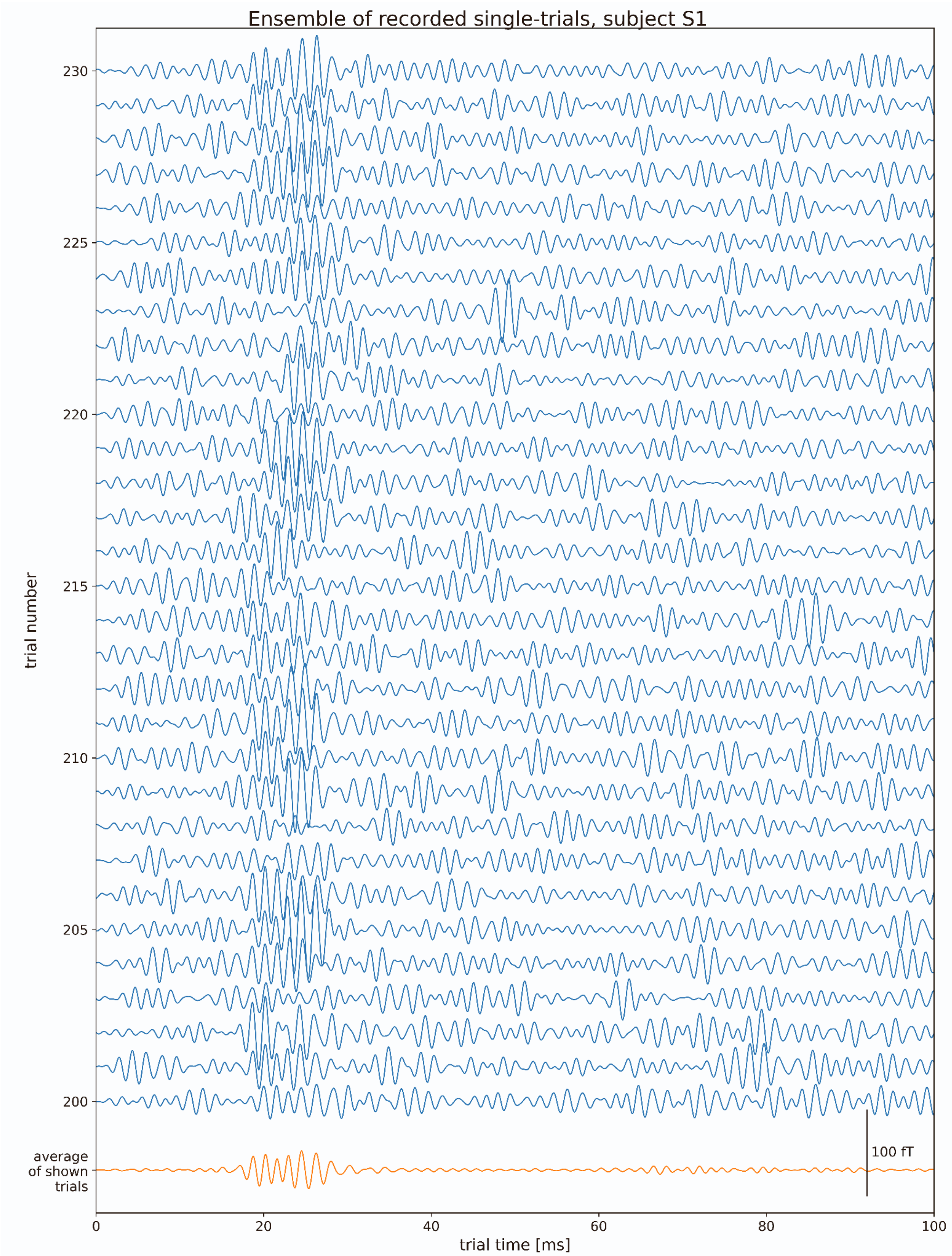

Fig. P1: Ensemble of subsequent single trials shown in chronological order (200-230) demonstrating their trial-to-trial variability as well as the relation to their average (all traces shown at identical scaling).

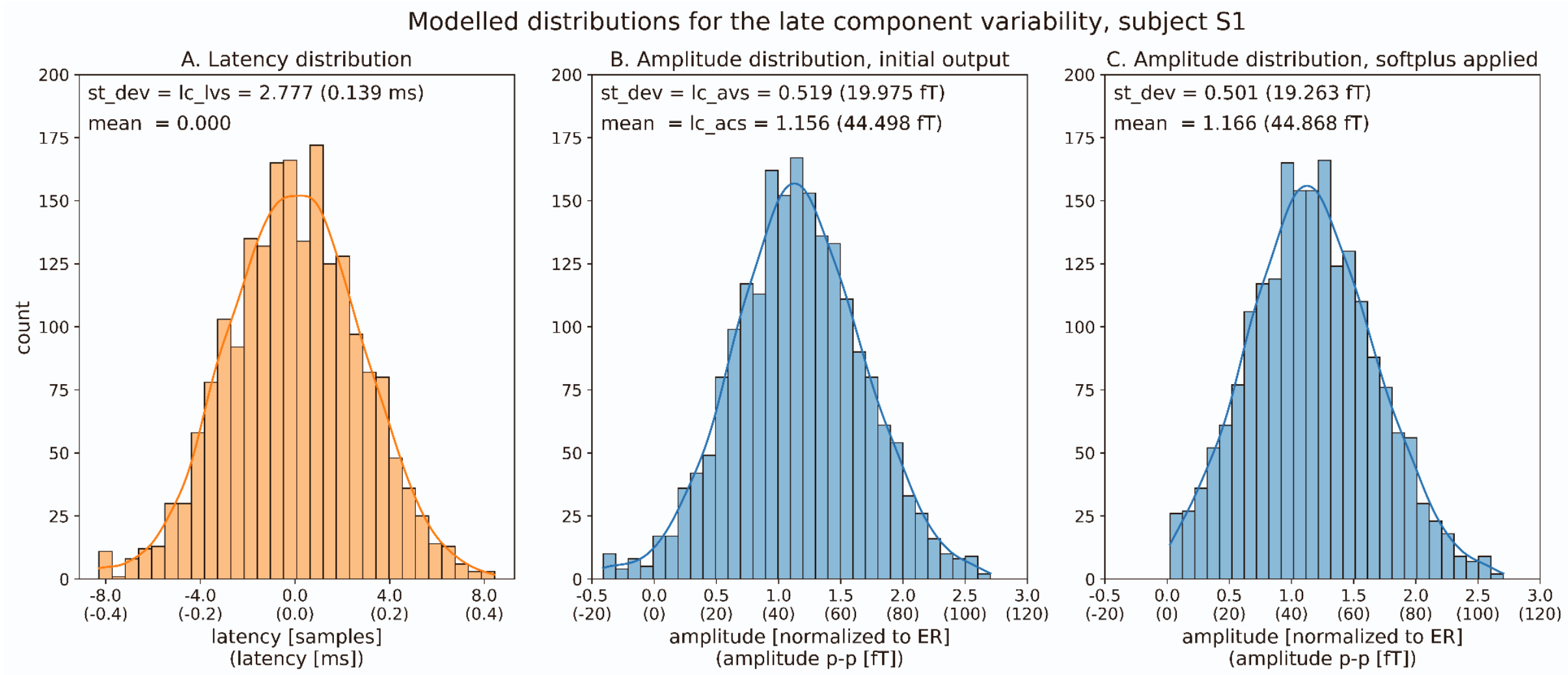

Fig. P2: Modelled distributions for the late component (4-parameter model), subject S1. A. latency distribution. B. Amplitude distribution, initial output. C. Amplitude distribution, softplus applied. In parentheses a corresponding physical dimension is shown.

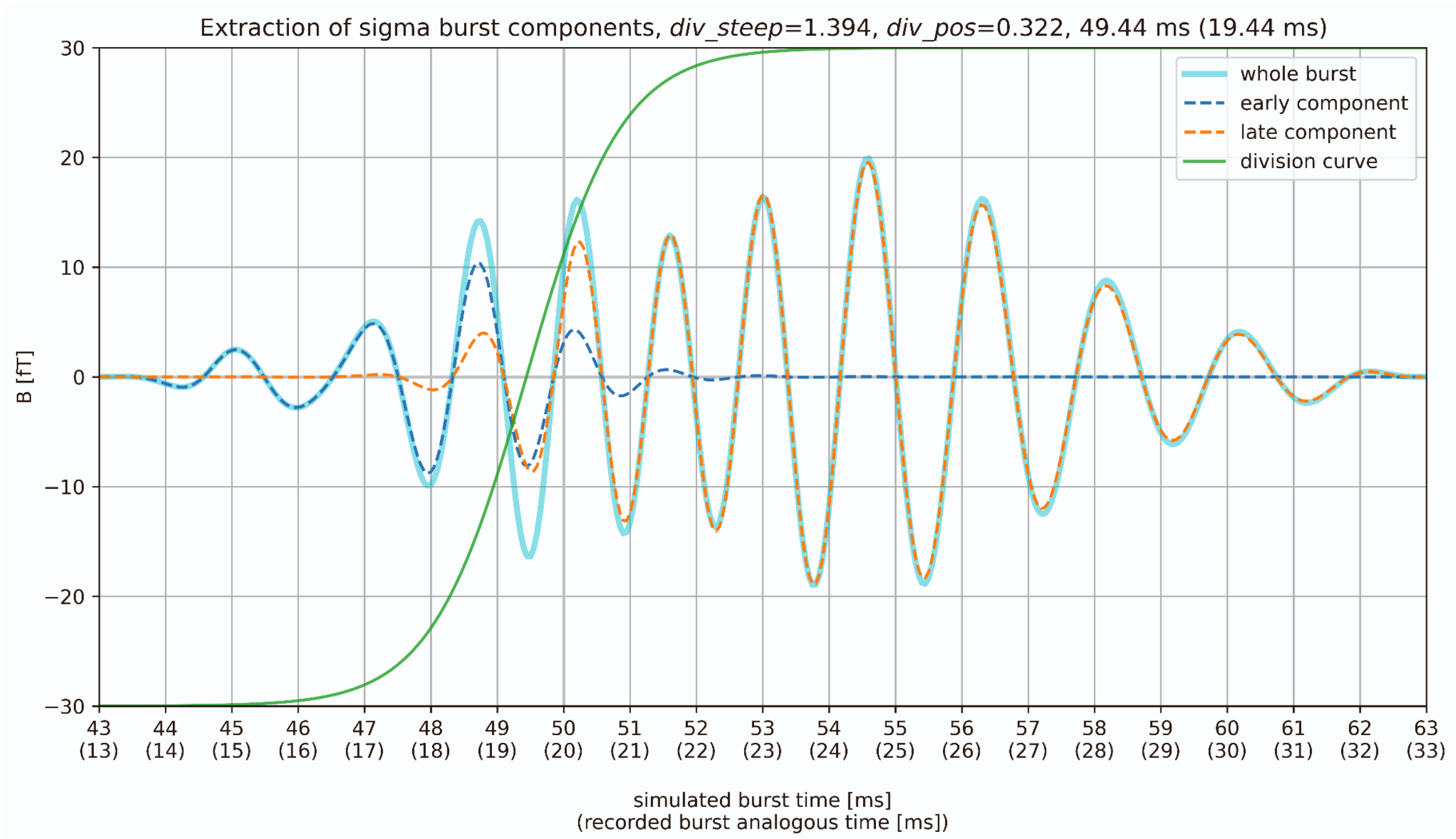

Fig. P3: Extraction of early and late burst components. Exemplary data from subject S1. As division curve, a sigmoid was used, the shape of which was controlled by two parameters, one for its steepness (*div_steep*) and one for its centre position (*div_pos*).

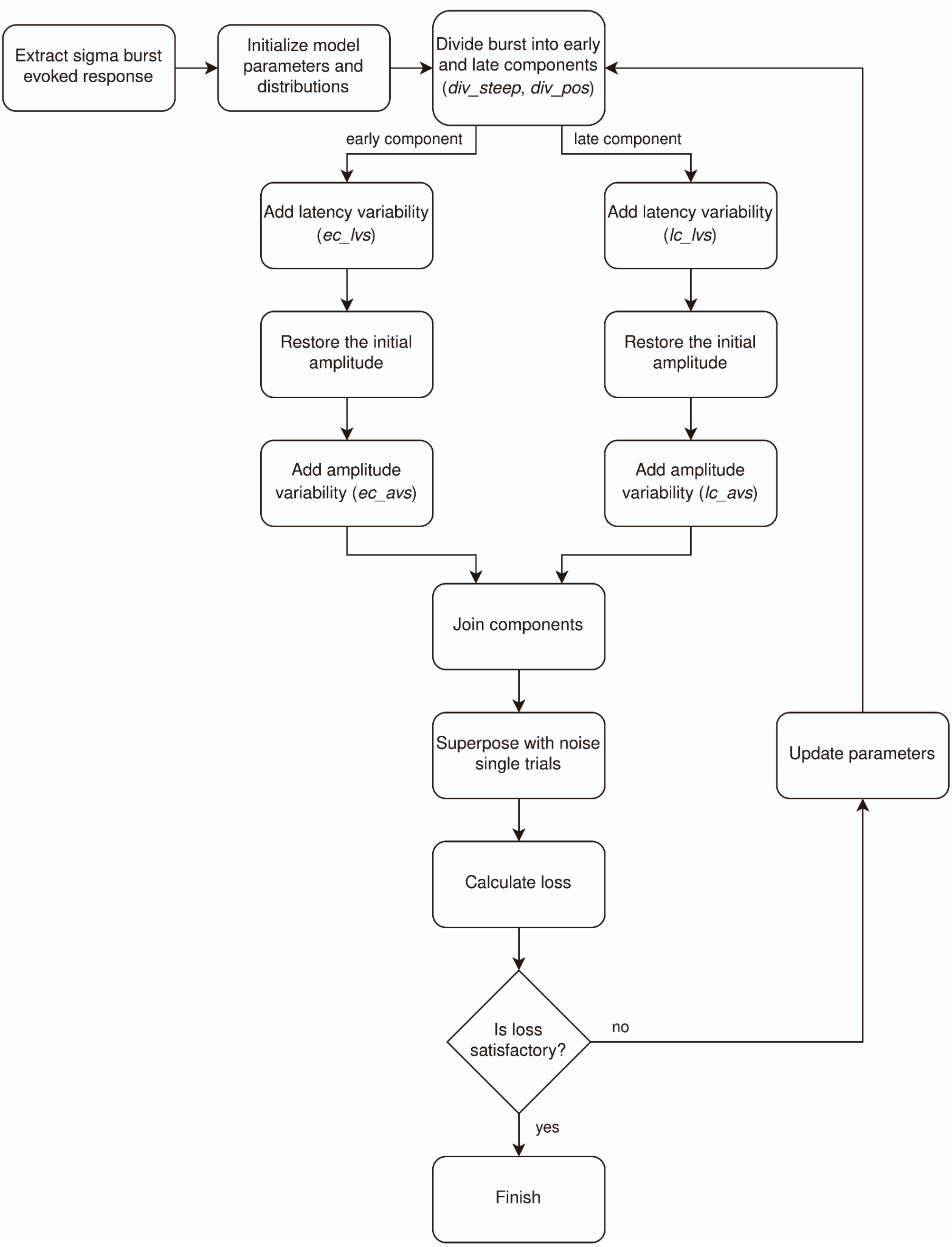

Fig. P4: Flowchart of the hfSERs modelling and parameter optimization process. The flowchart illustrates the sequential steps involved in the 6-parameter model optimization, parameters used at each step are indicated in parentheses. In simpler models, only part of the parameters are optimized, and others are set to constant values, e.g. *ec_lvs* and *ec_avs* are set to 0 in the 4-parameter model. The optimization process terminates when the calculated loss does not improve for a specified number of epochs.

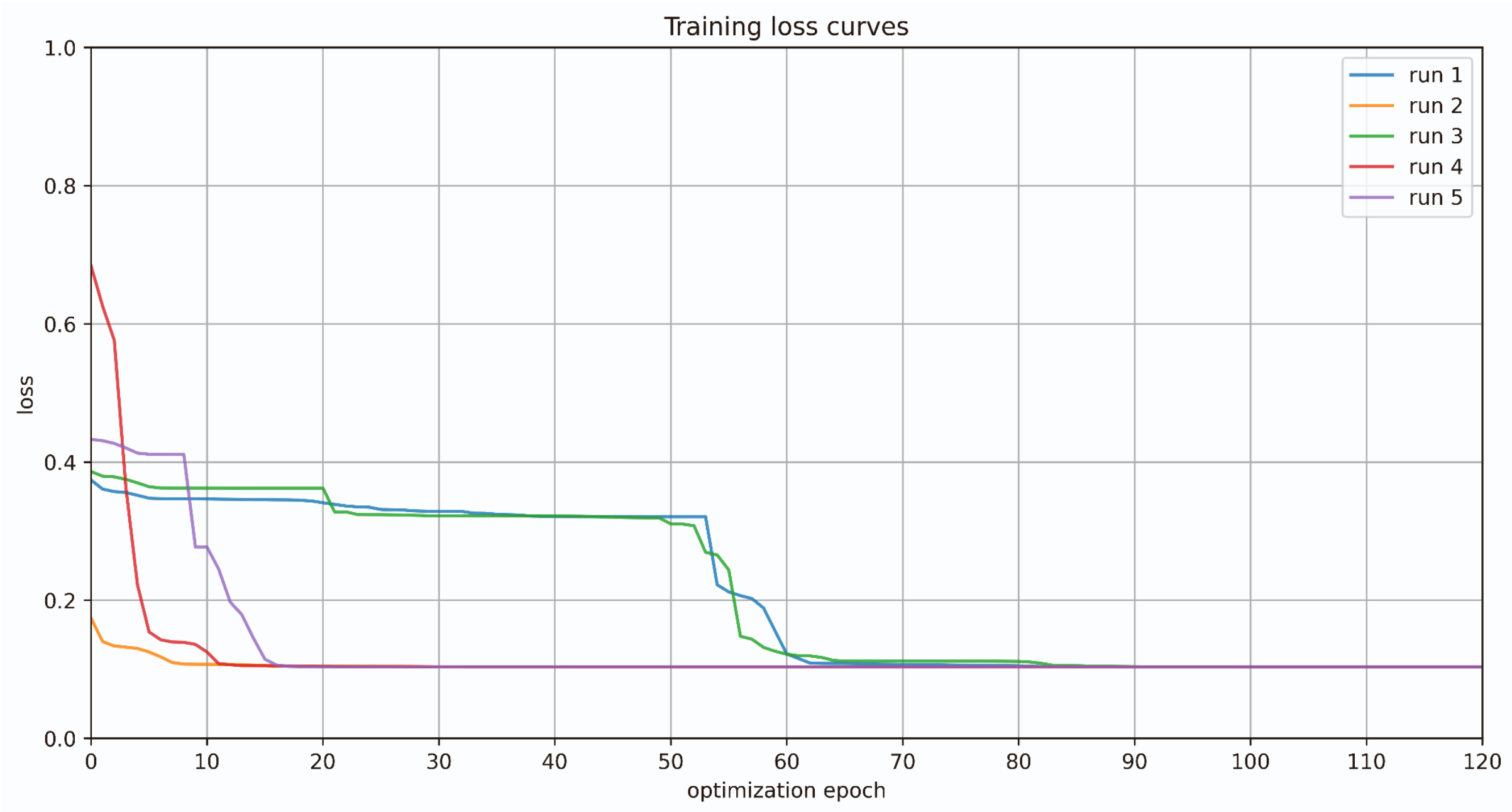

Fig. P5: Training loss curves for the model optimization. For each optimization, five independent runs were performed with each run starting at a different randomly chosen point to increase the chance of eventually finding the globally best solution. Most runs usually converged on the same minimum, which was presumably the global one.

Table 1: Results of optimization for model with different numbers of optimized parameters. Values presented in the table are losses calculated on the test set, values in parentheses indicate the percentage of variability explained by the model. If an outcome was worse than for the simpler model, the cell is marked in gray. The best result across subjects was achieved for the 4-parameter model, which was significantly better than the preceding, 2-parameter one ($p<0.01$).

**subject**	**number of optimized parameters**			
	**0**	**2**	**4**	**6**
S1	2.568 (0.0)	0.333 (87.0)	0.107 (95.8)	0.104 (95.9)
S2	1.541 (0.0)	0.470 (69.5)	0.273 (82.3)	0.277 (82.0)
S3	1.556 (0.0)	0.483 (69.0)	0.409 (73.7)	0.436 (72.0)
S4	1.908 (0.0)	0.392 (79.4)	0.299 (84.3)	0.339 (82.2)
S5	1.450 (0.0)	0.464 (68.0)	0.265 (81.7)	0.290 (80.0)
mean	1.805 (0.0)	0.428 (76.3)	0.271 (85.0)	0.289 (84.0)

Table 2: Results of optimization for 6-parameter model. Final values of the optimized parameters (*div_steep*, *div_pos*, *ec_avs*, *ec_lvs*, *lc_avs*, *lc_lvs*), training and test losses. Late component variability scalers (*lc_avs*, *lc_lvs*) showed to be significantly larger ($p<0.01$) than corresponding parameters of the early component (*ec_avs*, *ec_lvs*).

**subject**	***div_ steep***	***div_ pos***	***ec_avs***	***ec_lvs***	***lc_avs***	***lc_lvs***	**train loss**	**test loss**
S1	1.923	0.339	0.134	1.563	0.520	2.763	0.082	0.104
S2	3.589	0.342	0.036	0.529	0.253	1.393	0.192	0.277
S3	0.808	0.514	0.093	1.348	0.586	2.169	0.210	0.436
S4	1.649	0.560	0.151	1.456	0.756	3.156	0.177	0.339
S5	0.509	0.463	0.158	0.862	0.932	2.819	0.155	0.290
mean	1.702	0.444	0.119	1.152	0.609	2.460	0.163	0.289

Table 3: Results of optimization for 4-parameter model. Final values of the optimized late component parameters (*div_steep*, *div_pos*, *lc_avs*, *lc_lvs*), internally calculated *lc_acs* factor, training and test losses. Parameters for early component variability were set to 0 and not optimized.

**subject**	***div_ steep***	***div_ pos***	***lc_acs***	***lc_avs***	***lc_lvs***	**train loss**	**test loss**
S1	1.394	0.322	1.156	0.519	2.777	0.088	0.107
S2	2.163	0.326	1.043	0.252	1.396	0.198	0.273
S3	0.640	0.385	1.092	0.366	2.031	0.265	0.410
S4	0.818	0.354	1.112	0.483	2.140	0.202	0.299
S5	0.510	0.402	1.122	0.667	2.354	0.172	0.265
mean	1.105	0.358	1.105	0.457	2.139	0.185	0.271

Table 4: Frequency band and time window used to extract hfSERs in subjects.

**subject**	**hfSER frequency band [Hz]**	**hfSER time window [ms]**
S1	500-800	13-33
S2	500-800	13-28
S3	550-850	13-28
S4	550-850	13-25
S5	550-850	10-30

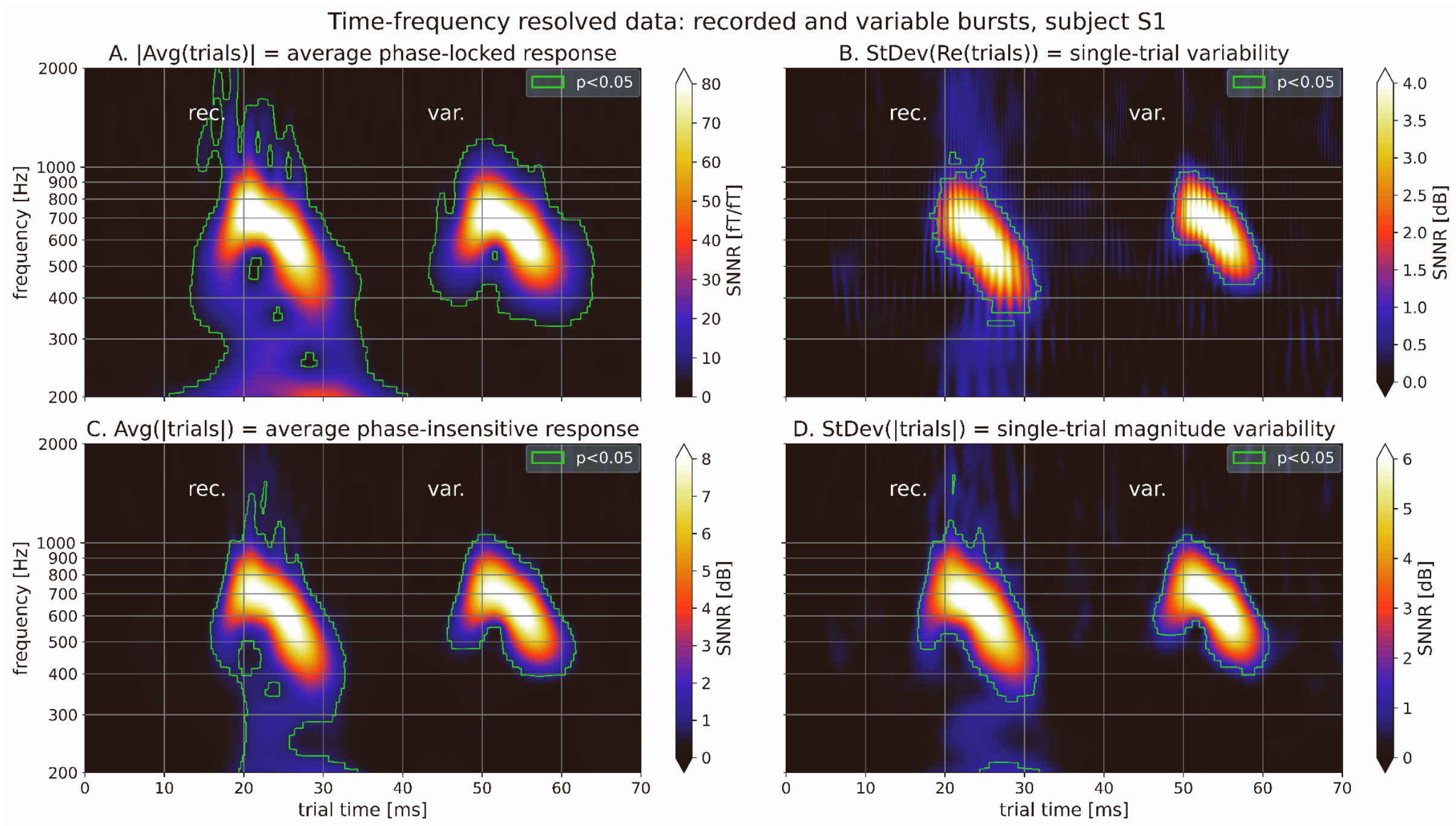

Fig. TF1: Analysis of time-frequency resolved data for subject S1.

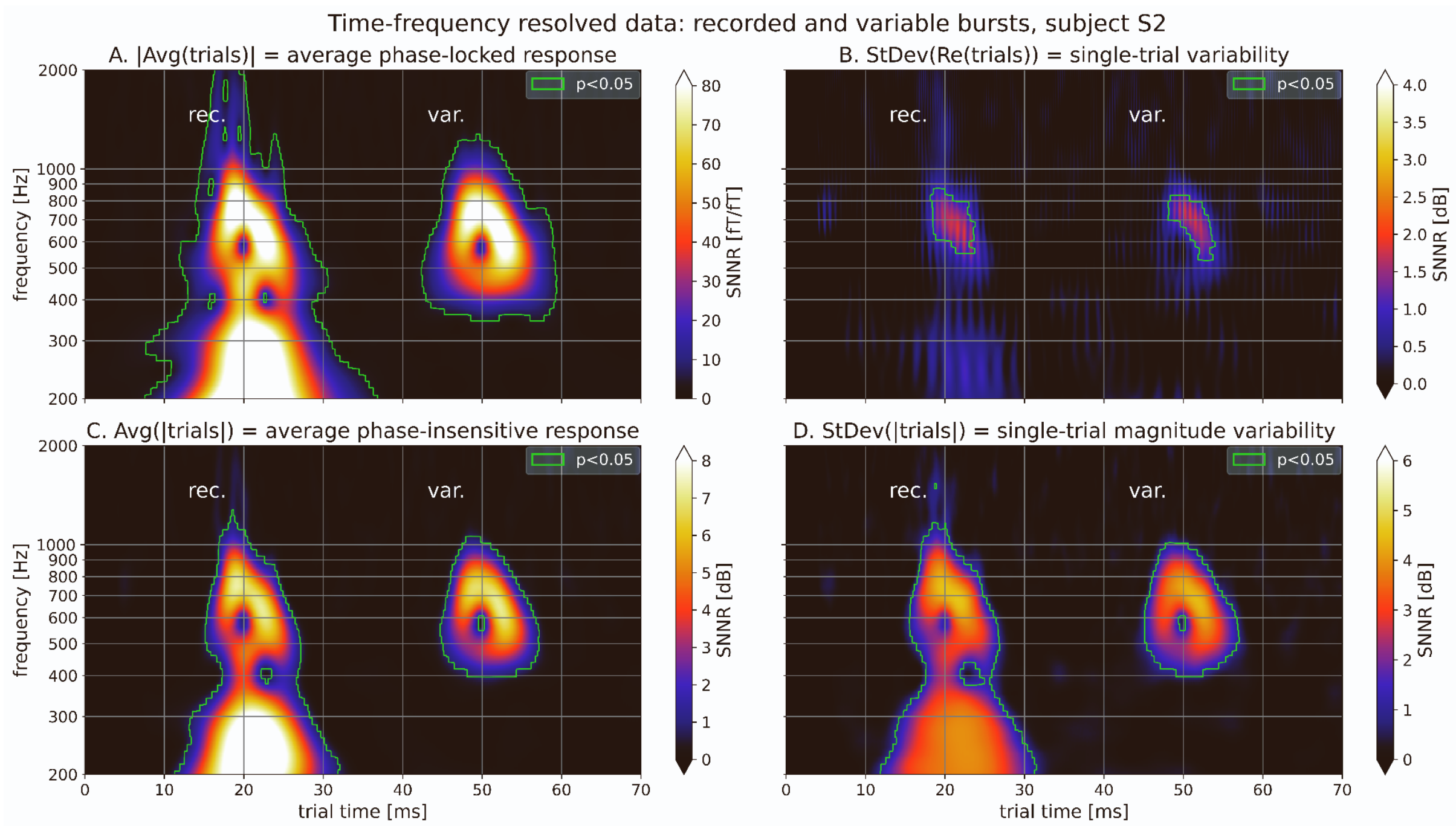

Fig. TF2: Analysis of time-frequency resolved data for subject S2.

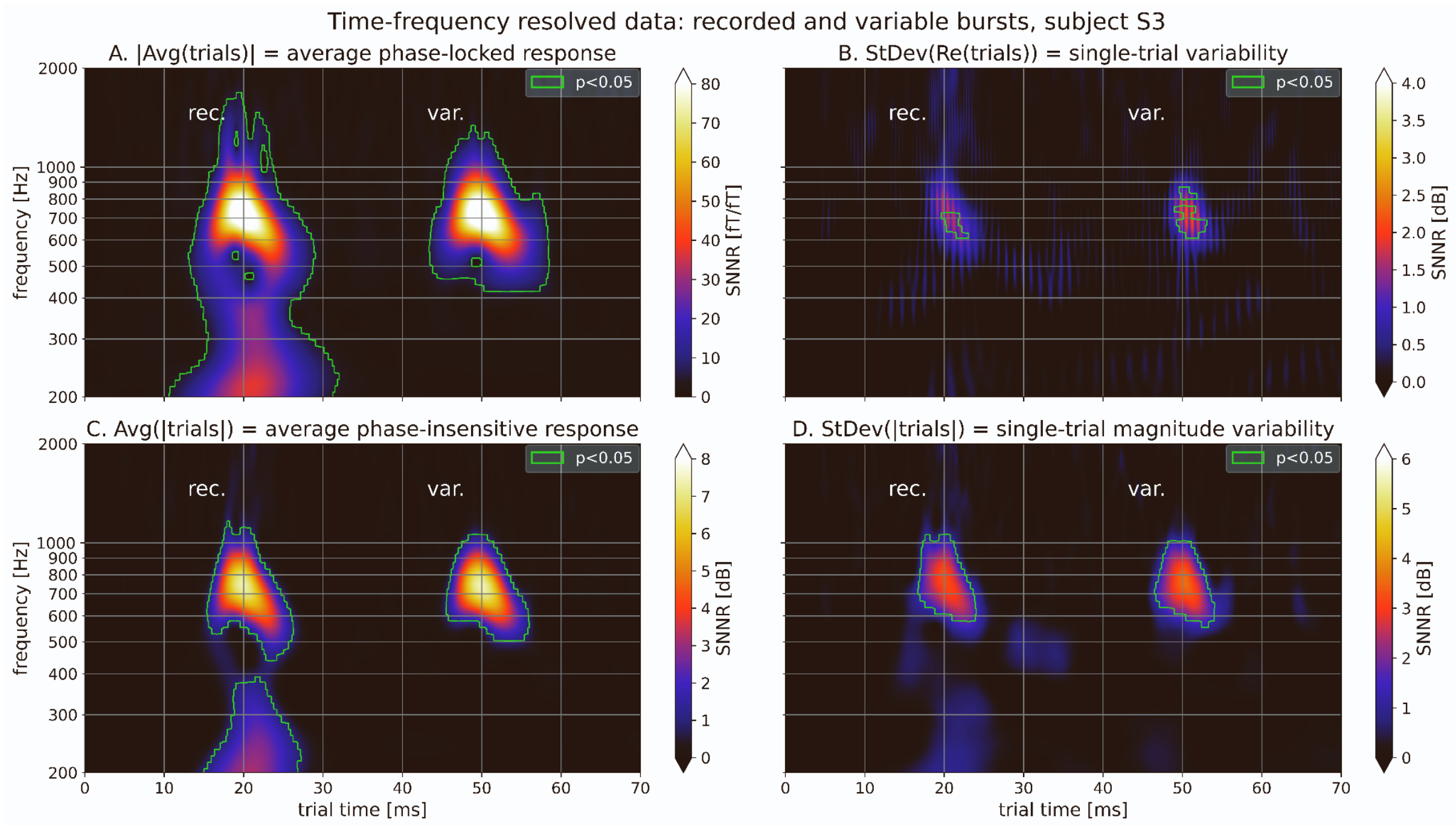

Fig. TF3: Analysis of time-frequency resolved data for subject S3.

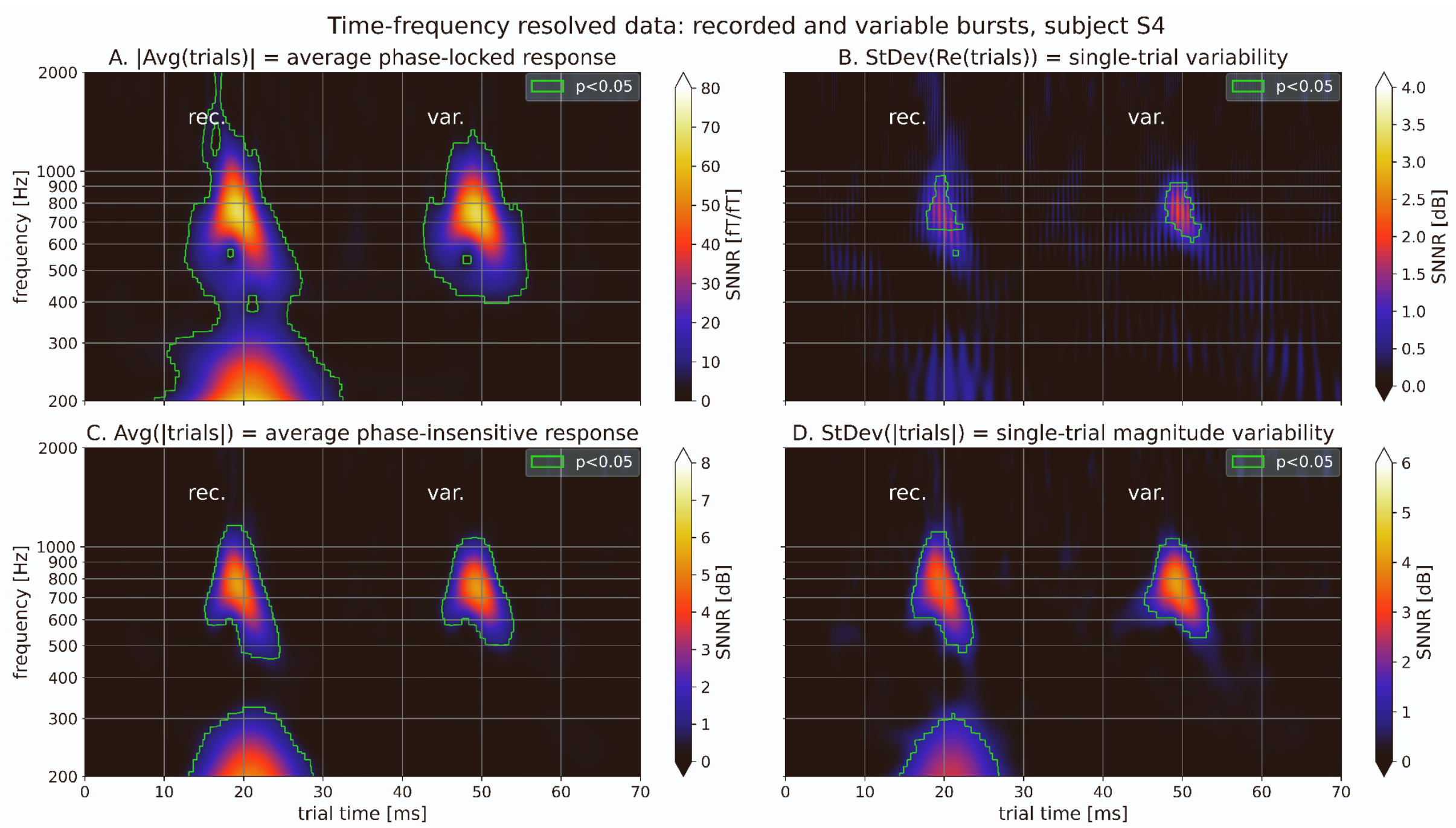

Fig. TF4: Analysis of time-frequency resolved data for subject S4.

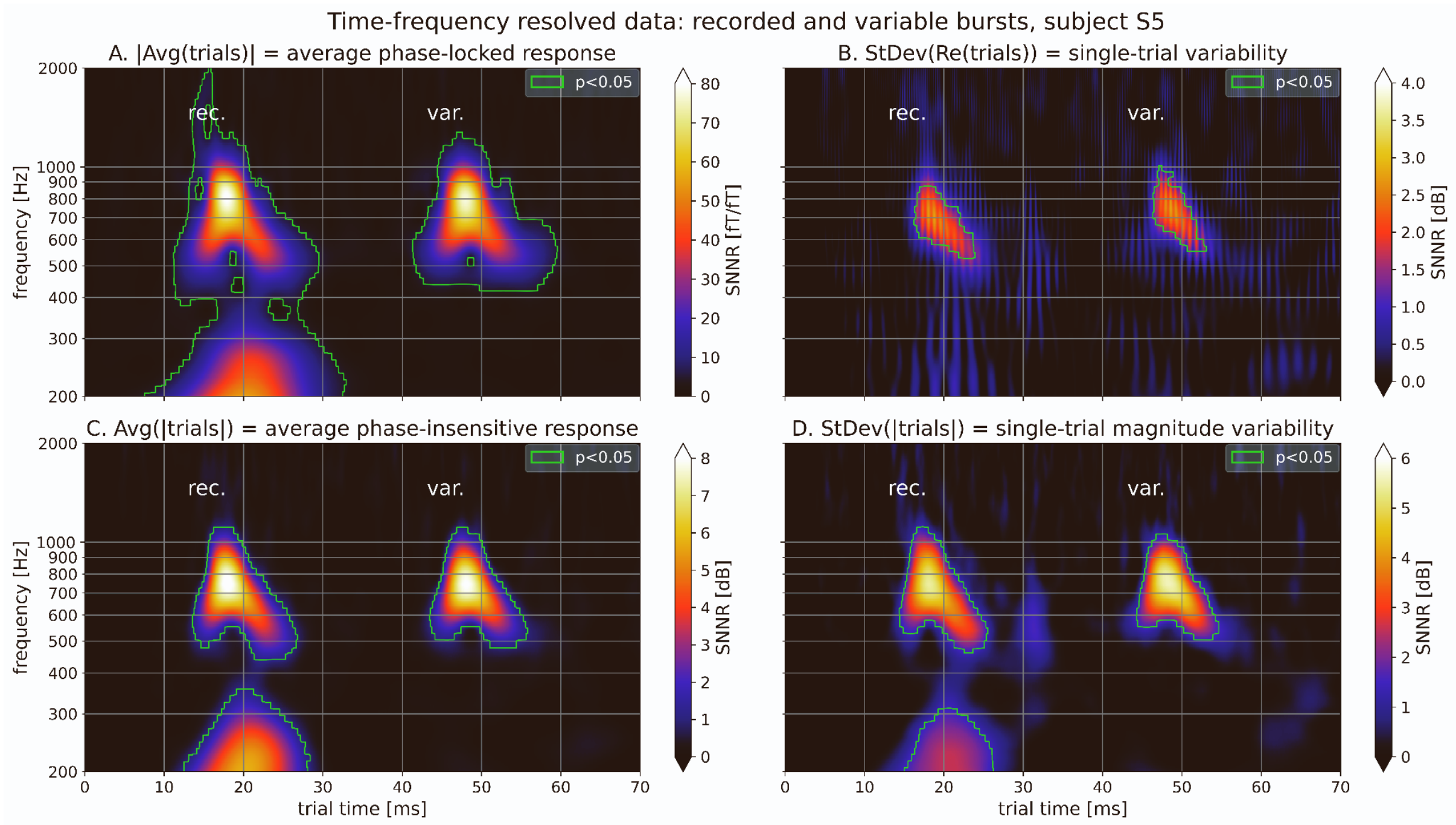

Fig. TF5: Analysis of time-frequency resolved data for subject S5.
